# Modified Atmosphere Packaging (MAP) for Seaweed Conservation: Impact on Physicochemical Characteristics and Microbiological Activity

**DOI:** 10.3390/foods12142736

**Published:** 2023-07-18

**Authors:** Bruno Moreira-Leite, Rafael Antunes, João Cotas, Nuno Martins, Nuno Costa, João P. Noronha, Paulina Mata, Mário Diniz

**Affiliations:** 1 LAQV-REQUIMTE, Department of Chemistry, NOVA School of Science and Technology, Universidade NOVA de Lisboa, Quinta da Torre, 2829-516 Caparica, Portugal; b.leite@campus.fct.unl.pt (B.M.-L.);; 2 Department of Life Sciences, Faculty of Science and Technology (FCTUC), Universidade de Coimbra, 3030-790 Coimbra, Portugal; jcotas@gmail.com; 3 MED—Mediterranean Institute for Agriculture, Environment and Development & CHANGE, Universidade de Évora, Pólo da Mitra, Apartado 94, 7006-554 Évora, Portugal; 4 UCIBIO-REQUIMTE, Department of Chemistry, NOVA School of Science and Technology, Universidade NOVA de Lisboa, Quinta da Torre, 2829-516 Caparica, Portugal; 5 Associate Laboratory i4HB, Institute for Health and Bioeconomy, NOVA School of Science and Technology, Universidade NOVA de Lisboa, 2819-516 Caparica, Portugal

**Keywords:** seaweeds, modified atmosphere packaging (MAP), food conservation, physicochemical characterization, microbiology, food safety

## Abstract

Conventional conservation techniques such as drying, salting or freezing do not allow for preserving the original characteristics of seaweeds. The present work aims to study the impact of minimal processing, in particular “Modified Atmosphere Packaging” (MAP), on the physicochemical characteristics and food safety of two seaweed species, “laver” (*Porphyra umbilicalis*) and “sea-lettuce” (*Ulva lactuca*), stored at 6 °C for 15 days. Different parameters were evaluated using analytical methods, namely the composition of headspace gases, color, texture, microorganisms, and volatile organic compounds (VOCs). The main findings of this study were that the MAP treatment was able to inhibit the respiration rate of minimally processed seaweeds, also preserving their color and texture. There was a remarkable reduction in the microbial load for *P. umbilicalis* treated under modified and vacuum atmospheres, and *U. lactuca* exhibited relatively steady values with no notable differences between the treatments and the control. Therefore, during the 15-day study period, both seaweeds met the requirements for food safety. GC–TOF-MS allowed to conclude that both MAP and vacuum treatments were more efficient in maintaining the odor characteristics of *U. lactuca* compared to *P. umbilicalis* with no significant differences throughout the storage days. Metabolic responses to diverse sources of abiotic stress seemed to account for most of the changes observed.

## 1. Introduction

Algae are uni- or multicellular, photoautotrophic organisms that live in aquatic or wet environments, with chlorophyll α as the primary photosynthetic pigment. Unlike plants, the reproductive cells do not have a sterile covering [1]. Macroalgae form a diverse group of macroscopic organisms that are distinguished by their size, color and morphology [2].

Macroalgae are an important source of biological resources that have been exploited in several research areas (biofuels, materials science, pharmacology, food and nutrition, etc.) [3]. Lately, there has been a growing interest in marine macroalgae (or seaweed), especially because of their importance as a source of new bioactive and functional compounds [4]. Most of the published research deals with the nutritional or health benefits that edible seaweeds can promote in food formulations, rather than valuing them as an ingredient with gastronomic potential [5].

Marine macroalgae are rich in protein and dietary fiber, an excellent source of vitamins, trace elements and minerals, and are low in sugars and lipids, with a predominance of polyunsaturated fatty acids—ω-3 (EPA and DHA) and ω-6 (AA) types—in a balanced ratio. These nutritional characteristics make seaweeds wholesome ingredients and low in calories [2]. There is also a wide variation in the chemical composition of marine macroalgae of the same genus, resulting in different contents of the main nutrients (macro- and micronutrients), depending on seasonal, climatic and geographic conditions [6].

Despite seaweed’s numerous nutritional benefits, its role in the diet of the Western world remains insignificant. An increasing interest in its use as food can be explained by a growing awareness of the Western public about its possible health benefits [7,8]. Nevertheless, developing novel products and recipes in partnership with talented chefs or adapted to local cuisines could boost even more seaweed consumption in these countries [9].

The United Nations estimates that the population should approach 9.7 billion in 2050. In an increasingly populated planet, there is an emerging concern about the sustainability of the existing means of production and food resources [10]. In light of this, seaweeds with the potential for use in food have emerged as a viable strategy to meet the increase in demand for food, in particular due to their high protein content and low or negative carbon footprint [11].

Conservation techniques have the function of preserving the quality of food, from the moment of production to its consumption. They prevent microbial growth and physicochemical changes, thus allowing for the maintenance of organoleptic qualities and increasing shelf-life [12]. Processing methods can also be used for other purposes, such as transforming the flavor through salting, curing or smoking, or even making foods more nutritious and digestible through fermentation or cooking [13]. They also help to overcome the issue of seasonality for certain foods, allowing for their consumption throughout the year, as well as maintaining a more diverse diet [12]. Marine macroalgae are perishable ingredients that undergo many physicochemical changes as soon as they are removed from their environment. Moreover, according to the conservation methods applied, the changes can be even more noticeable due to the action of physical (e.g., heat or solar UV radiation), chemical (e.g., pH change or addition of salt) and/or biological (e.g., enzymatic reactions or action of microorganisms) factors [14]. Preservation techniques applied so far in the processing of seaweeds (such as drying, salting or freezing) do not allow for keeping their organoleptic and nutritional properties unchanged [15,16].

Drying can lead to a loss of volatile organic compounds (VOCs) and the emergence of off-flavors due to the auto-oxidation and enzymatic reactions [17,18]. Furthermore, during the entire process, the product is subjected to temperatures higher than the glass transition temperature (T*g*), which results in shrinkage of the tissues [19].

Salting has less pronounced effects on VOCs due to its ability to inhibit oxidation reactions by applying low processing temperatures and promoting osmotic dehydration. However, the “salting out” effect can also lead to a loss of volatiles and the alteration of sensory properties such as firmness [20,21,22]. Both drying and salting techniques are based on water activity (a_w_) reduction.

The major drawback of freezing stems is the loss of texture (decreased hardness), due to ice crystal formation that damages seaweed tissues [23,24]. The type and content of hydrocolloids present in the seaweeds will also affect water loss—and consequently phytochemical composition—during long-term frozen storage [25]. A study also revealed that frozen marine macroalgae tend to develop green aromas, probably as a consequence of the auto-oxidation and enzymatic conversion of aldehydes from polyunsaturated fatty acids via the action of lipoxygenases (LOX) [22,26,27].

In addition, the loss of nutritional value, water-holding capacity alteration, and significant color modification are also reported as being the main changes promoted by using conventional conservation techniques such as drying, salting or freezing [15,28].

Freeze-drying processing can overcome some of the aforementioned problems, but it has the disadvantages of high costs and long processing times, being used only for products with high added value [29,30,31]. Modern techniques such as high-pressure processing (HPP) still exhibit high operating costs, being applied to seaweeds only in scientific trials. In the tests performed, HPP showed to be a valid processing technology for extending the shelf-life of edible seaweeds under refrigerated storage; although, microbiological, physicochemical, color, and texture attributes along with enzymatic activity, varied significantly between treatments and storage time [32,33].

Modified atmosphere packaging (MAP) is a technique used to increase the shelf-life of fresh or minimally processed foods. In this conservation method, the air that surrounds the food in the package is suppressed and substituted for a gas (or mixture of gases) with another composition. This new atmosphere mainly acts by modulating the respiration of fresh products, and by inhibiting microbial activity and oxidation. The gas mixture in the package depends on the type of product, packaging materials and storage temperature. MAP allows the original characteristics of the products to be extended [34].

Despite its widespread use in vegetables, meat, and other foods, there is currently no known article discussing the utilization of MAP for preserving seaweed. Paull & Chen [35], however, studied the use of vacuum-packing as a preservation method for *Gracilaria salicornia* (Rhodophyta). The main remarks by these authors were that reducing the moisture content of the storage container led to seaweed discoloration and cellular leakage. Additionally, vacuum storage to lower the air content did not result in an extended post-harvest shelf-life. Containers and wraps with high gas exchange rates were found to be less effective in prolonging shelf-life. However, seaweed that was fully immersed in seawater and kept in the dark at 17 °C remained viable and free from discoloration for over a month [35].

The present work aims to study the impact of MAP on the physicochemical characteristics and food safety of two seaweed species: “laver” (*Porphyra umbilicalis,* Rhodophyta) and “sea-lettuce” (*Ulva lactuca,* Chlorophyta). For this purpose, a mixture of gases—consisting of 80% argon (Ar) and 20% carbon dioxide (CO_2_)—together with vacuum packaging were used. CO_2_ was selected due to its antimicrobial properties and its role as a carbon source for seaweed’s metabolism. Argon, on the other hand, was chosen for its nitrogen-like properties, along with its superior water solubility and ability to inhibit specific enzymatic reactions. Physical, microbiological, and chemical parameters were evaluated: composition of headspace gases (respiration rate), color, texture (hardness), pathogenic bacteria and marine heterotrophic bacteria counts, and volatile organic compounds (VOCs) through gas chromatography–time-of-flight mass spectrometry (GC–TOF-MS).

## 2. Materials and Methods

### 2.1. Materials

#### 2.1.1. Raw Materials

**Figure 1:**
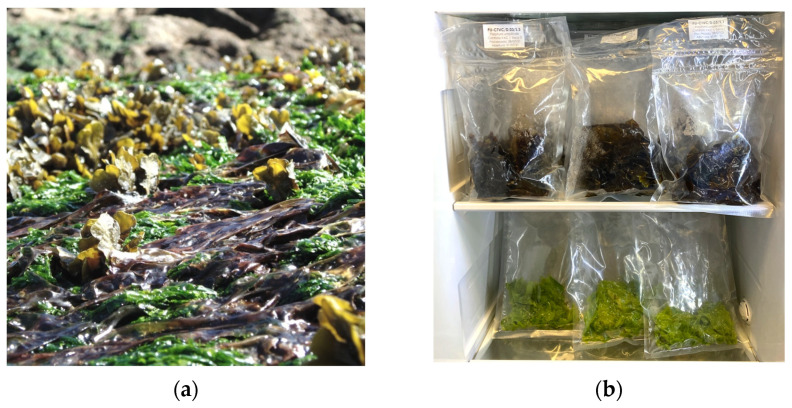
(**a**) *Ulva lactuca* (vivid green) and *Porphyra umbilicalis* (brownish purple) seaweeds exposed during low tide; (**b**) Seaweeds after processing (Controls and MAP treated samples).

*Porphyra umbilicalis* was collected at Praia da Tamargueira, Buarcos, Figueira da Foz (40°10′18.6″ N, 8°53′44.4″ W), Portugal. Samples were collected from areas with well-established macroalgae specie patches and no obvious epiphytes or deterioration, during the morning low tide (Figure 1a), being packed in plastic bags and kept in thermal boxes. The specimens were transported to the Marine Algae Laboratory (Dep. Life Sciences) at the University of Coimbra, where they were washed with filtered seawater (harvested at the same location) to remove sand and macroscopic epiphytes. The samples were selected by hand, discarding the non-standard specimens, and placed on sieves to remove excess moisture. Then, they were weighed (300 g), stored in plastic bags under partial vacuum (~80%), and kept under refrigeration (8 °C). After 24 h, the samples were transported in thermal boxes to Faculty of Science and Technology (FCT NOVA), where they were processed.

Due to *Ulva lactuca* being an estuarine macroalgae, it was cultivated on a prototype system developed by Lusalgae Lda. (Figueira da Foz, Portugal) and the Marine Algae Laboratory (Dep. Life Sciences) at the University of Coimbra.

The method was based on Araujo et al. [36] in which *U. lactuca* was grown in a water tank exposed to direct sunlight with aeration during the day (normally 14 h) with the goal of obtaining enough biomass for the assays. To minimize differences in biochemical composition caused by the macroalgae growth condition, the cultured species were identical to those of the indoor culture described by García-Poza et al. [37]—i.e., collected in the same pools and dates, with a length of less than 5 cm. As a result, the culture technique was the primary factor affecting the biochemical profile.

The culture medium was estuarine saltwater (23–34 PSU) taken from the Mondego River estuary in Figueira da Foz, Portugal, with no fertilizers added. The culture tank was 1000 L in volume and contained 800 L of mechanically filtered estuarine seawater [32].

The cultivation began with an initial amount of 600 g of wet biomass in a single tank. Three times each week, about 75% of the amount of water in the tank was changed, and after three weeks, all the biomass was collected for subsequent examination.

#### 2.1.2. Sample Processing

Seaweeds of the species *Porphyra umbilicalis* and *Ulva lactuca* were weighed (30 ± 1 g) and placed in 300 × 400 mm polyamide/polyethylene (PA/PE) bags with 90 μm thickness (Sammic, Azkoitia, BC, Spain), with this procedure being performed in triplicate for each day and treatment (Figure 1b). Using a vacuum machine SU-316 (Sammic, Azkoitia, BC, Spain), with an inlet for inert gases, the samples were submitted to three treatments: control samples (CTRL) were prepared by sealing the packages and keeping the ambient air inside the bags; vacuum-packed samples (VAC) were prepared by removing the air until the pressure reached 5 mBar inside the chamber; “Modified Atmosphere Packaging” samples (MAP) were prepared by removing the air and refilling 80% of the headspace with the ALIGAL^®^ 62 gas mixture (80% Ar + 20% CO_2_) (AirLiquide, Paris, IdF, France).

To simulate the most common retail storage conditions, all samples were stored at 6 ± 2 °C in a refrigerator (Model GSL360ICEV, LG, Seoul, South Korea), where a LED lighting system was installed and regulated to provide an average of 1.5 ± 0.2 W·m^−2^ in cycles of 12 h of light and darkness [38,39]. The lighting was carefully controlled, with a timer that provided light from 9 h to 21 h, which is consistent with the opening times of most supermarkets in Portugal. We also registered the light intensity in refrigerators (salads preserved with MAP) across five different stores and calculated the average intensity to replicate the conditions in the laboratory.

The tests lasted 15 days, and on days 3, 6, 9, 12, and 15, the samples were removed from the refrigerator and processed according to predefined analysis protocols. Before opening the packages, the composition of headspace gases was analyzed. Then, 12 seaweed thalli were randomly selected for color and texture measurement. The samples for the microbiological analysis were emptied inside the vacuum machine and then sealed to avoid ambient contamination. The material not analyzed on the same day was immediately frozen and kept at −45 °C until further analysis.

### 2.2. Composition of Headspace Gases

The internal O_2_ and CO_2_ composition of the packages containing the gas mixture (MAP) along with CTRL were monitored on days 3, 6, 9, 12, and 15 of the study by using a Dansensor CheckPoint Gas Analyzer (AMETEK-Mocon, Brooklyn Park, MN, USA) to examine 10 mL of the headspace of three true replicates [40]. As there was no headspace for the VAC samples, the analyses were not performed for this treatment.

### 2.3. Physical Characterization

#### 2.3.1. Color Measurement

A colorimeter model CSM-4 (PCE Instruments, Tobarra, C-L.M., Spain) was used to measure the CIELAB color coordinates. The CIELAB is a three-dimensional color space that represents color information in terms of lightness (L*), red–green axis (a*), and blue–yellow axis (b*).

Color deviation from the standard (∆E) was calculated using Equation (1):

(1) $$∆E_{t,d}=\sqrt{{∆L_{t,d}}^{2}+{∆a_{t,d}}^{2}+{∆b_{t,d}}^{2}}\;\mathrm{or}\;∆E_{t,d}=\sqrt{{\left(L_{t,d}-L_{0}\right)}^{2}+(a_{t,d}-a_{0}{)}^{2}+{\left(b_{t,d}-b_{0}\right)}^{2}}$$

where $L_{0}$, $a_{0}$ and $b_{0}$ are the averages of the readings for the coordinates ($L^{*}$, $a^{*}$, $b^{*}$) made at time zero for the control, i.e., before the seaweeds undergo any treatment or storage; $L_{t,d}$, $a_{t,d}$ and $b_{t,d}$ are the readings taken after processing (*t* = CTRL, MAP or VAC) and storing the samples (*d* = 3, 6, 9, 12, or 15 days) [41].

The parameters for the readings were aperture 20 mm, illuminant D_65_, and geometry 45°/0° (illumination/viewing angle). The equipment was calibrated for white before collecting the data and the readings were taken immediately after the samples were removed from the packages. For each sample, 12 replicates were measured against a white canvas, and the averages for each coordinate were calculated.

#### 2.3.2. Texture Analysis

Due to the morphology of the seaweeds, which is characterized by the presence of laminar thalli resembling a film, a rupture test, which analyzes the force to break the sample’s tissue, was chosen to evaluate the texture. The hardness of the species was measured using a CT3 Texture Analyzer with a load cell of 45 N (Brookfield, New York, NY, USA). The samples removed from bags were randomly chosen, cut into circles (⌀ 35 mm) using a metal cutter, and placed in Petri dishes with seawater. A base table (Model TA-BT-KIT), having attached a fixation support (200 × 200 × 10 mm, acrylic, hole ⌀ 7.65 mm), was used to place the samples that were punctured using a cylindrical metal probe with ⌀ 4 mm (TA-44). The tests were performed at room temperature (22 ± 2 °C). All the samples were removed from seawater 5 min before the analysis and placed between 2 sheets of filter paper to remove excess moisture. Data acquisition was conducted at the rate of 10 points/s with a trigger load of 20 mN and a correction of 200 mN (discharge) using TexturePro CT Software (Brookfield, New York, NY, USA). The pre-test speed was set to 2 mm/s, the test speed to 0.1 mm/s, and the post-test speed to 4.5 mm/s. The maximum force was measured by making one puncture in each fresh alga, using 10 samples per day and treatment. The average values were then calculated and the results were expressed in N [42,43].

### 2.4. Microbiological Analysis

To avoid contamination, samples were prepared in a horizontal laminar airflow cabinet (Aeolus H, Telstar^®^, Terrassa, Cat., Spain). A total of 10 g of each sample were weighed and placed aseptically into a sterile blender bag (BagLight PolySilk, Interscience, Saint-Nom-la-Bretêche, IdF, France) and homogenized with 90 mL of quarter-strength Ringer’s solution (Biokar Diagnostics, Allonne, Oise, France) at room temperature. After 90 s in a stomacher apparatus (BagMixer^®^ 400 P, Interscience, Saint-Nom-la-Bretêche, IdF, France), appropriate serial dilutions were spread-plated or dispersed by the pour plate method, according to the protocol, in Petri dishes Ø90 mm (Frilabo, Oeiras, PEs, Portugal). Only plates containing at least 10 colony-forming units (CFUs) and up to 300 characteristic and/or non-characteristic CFUs were considered for each dilution. If the values were below or above this range, they were reported to be <LOQ (limit of quantification) or >3.0 × 10^5^ CFU·g^−1^ (5.4771 log CFU·g^−1^), respectively.

*Escherichia coli*, coagulase-positive Staphylococci and *Vibrio* spp. Were quantified according to the methods described in ISO 16649-2, ISO 6888-2 and ISO 21872, respectively [44,45,46]. For the detection of *Salmonella* spp. And *Listeria monocytogenes*, the methods described in ISO 6579 and ISO 11290-2, respectively, were used [47,48]. Total coliforms were quantified according to an internal method based on the technical datasheet for the Compass^®^ Ecc Agar [49], and heterotrophic marine bacteria (HMB) were quantified according to an internal method based on the datasheet instructions for the Condalab Marine Agar [50].

There is an absence of comprehensive European regulations specifically addressing seaweed. However, France is one of the few countries that has established regulations in this regard, defining a limit of 10^5^ CFU·g^−1^ (5 log CFU·g^−1^) for aerobe mesophiles (30 °C) in products containing dried seaweed [51]. Furthermore, Portuguese legislation sets the limit for “minimally processed” fresh products at 10^6^ CFU·g^−1^ (6 log CFU·g^−1^) [52]. Therefore, the Intermediate value of 5.4771 log CFU·g^−1^ was adopted as the threshold for the safe consumption of the studied seaweeds.

### 2.5. Chemical Characterization

#### GC–TOF-MS Analysis

A carboxen/divinylbenzene/polydimethylsiloxane (CAR/DVB/PDMS) fiber for autosamplers—1 cm, 50/30 μm film thickness (d_f_) (Supelco, St. Louis, MO, USA)—was used for HS-SPME extractions. Fiber blanks were run periodically to ensure the absence of contaminants and/or carryover. The thalli from the seaweeds were removed from three different packages (the same ones for which the composition of headspace gases was measured), combined and ground using liquid nitrogen. HS-SPME extraction was performed according to the following procedure: 3 g of each fresh seaweed sample were introduced in a 20.0 mL headspace vial and sealed with a PTFE/silicone septum and a magnetic screw cap (Supelco, USA). The vial was equilibrated for 10 min at 50 °C and then extracted for 30 min at the same temperature. Thermal desorption of the analytes was carried out by exposing the fiber in the GC injection port at 260 °C for 3 min in splitless mode.

The analyses were performed on a GC–TOF-MS system consisting of a GC 8890 System (Agilent Technologies, Santa Clara, CA, USA) with a BenchTOF-Select detector (Markes International, UK). An automatic sampler injector—CTC Analysis autosampler PAL-System (SepSolve Analytical, Peterborough, Cambs, England, UK)—was used and the data were acquired and analyzed with TOF-DS 4.1 (Markes International, Bridgend, MGM, Wales, UK). Chromatographic separation was achieved on a DB-WAX (60 m × 0.25 mm I.D. × 0.25 μm d_f_) polyethylene glycol capillary column (Agilent Technologies, Santa Clara, CA, USA). The oven temperature program began at 50 °C, held for 2.5 min, raised at 3 °C·min^−1^ up to 90 °C, at 6 °C·min^−1^ up to 140 °C, 2 °C·min^−1^ up to 180 °C and finally 20 °C·min^−1^ up to 240 °C and held for 20 min to ensure column cleanliness [22]. The MS transfer line and source temperatures were set at 250 °C. Helium was used as the carrier gas.

Spectra were matched using the NIST MS Search Program, version 2020. To determine the retention times and characteristic mass fragments, electron impact ionization (EI) at 70 eV was used and mass spectra of the analytes were recorded at full scan, from 30 to 400 Da. The linear retention index (LRI) values were calculated through the analysis of an *n*-alkane standard solution (C8–C20) (Supelco, St. Louis, MO, USA), using the same chromatographic conditions [53]. The volatile compounds were first identified by matching mass spectra with the spectra of reference compounds in the NIST mass spectral library, also taking into consideration structure and molecular weight, and by comparing the calculated LRIs with those described in the literature. Additionally, 37 analytical standards (Sigma-Aldrich/Merck, Darmstadt, HE, Germany) were diluted to 100 ppm in hexane PA and 1 μL was injected, using a 1:20 split rate and the same ramp program, to make a more accurate identification of the main volatile organic compounds (VOCs) present in the seaweeds. The relative amount of each compound was calculated as the percent ratio of the respective peak area relative to the total peak area of the chromatogram and expressed as a percentage (%).

### 2.6. Statistical Analysis

Statistical analyses were performed with RStudio version 2022.2.1.461 [54] using fully randomized two-way ANOVA followed by the Tukey honest significant difference (HSD) *post-hoc* test. All data series residues were tested for normality (Shapiro–Wilk test), symmetry (D’Agostino test), kurtosis (Anscombe–Glynn test), and homoscedasticity (Levene test). Results were expressed as mean ± standard deviation (SD), except for the color analysis, for which results were expressed as median ± interquartile range (IQR). Given the nature of the data, the color parameters and the GC–TOF-MS peak relative areas were analyzed using non-parametric tests according to the methodology proposed by Elkin et al. [55]: aligned rank transformation (ART) ANOVA to verify the significant differences, followed by pairwise comparison using the ART contrasts (ART-C), both available in the “ARTool” package for RStudio. The statistical significance reference was α = 5% for all the analyses. To identify which VOCs contributed most significantly to the variation of the samples in terms of volatiles, principal component analysis (PCA) was carried out using the statistical software The Unscrambler^®^ X, version 10.5.46461.632 (CAMO Software AS, Oslo, OEST, Norway).

## 3. Results

### 3.1. Composition of Headspace Gases

**Figure 2:**
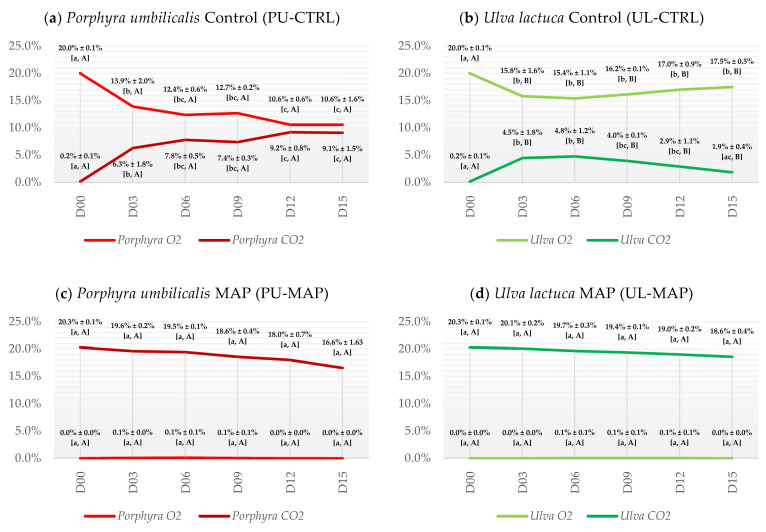
The composition of headspace gases for O_2_ and CO_2_ throughout storage days for *P. umbilicalis* (**a**,**c**) (left column) and *U. lactuca* (**b**,**d**) (right column) controls (**a**,**b**) (upper line) and MAP-treated seaweeds (**c**,**d**) (lower line). Notes: The results are expressed as % of the headspace composition. Means ± SD from triplicate determinations on each experiment. Means followed by a different lowercase letter are significantly different (*p* < 0.05) throughout storage days. Means followed by a different uppercase letter are significantly different (*p* < 0.05) between seaweed species (same treatment and gas).

As shown in Figure 2, the oxygen (O_2_) concentration for the control (CTRL) and carbon dioxide (CO_2_) concentration for the MAP treatment, for both seaweeds, tended to decrease over time. However, no significant differences were found for the O_2_ or CO_2_ concentrations (i.e., respiration pattern) over time for the MAP treatment for either seaweed (Figure 2c,d). For CTRL, the headspace composition for each seaweed differed significantly after day 0 for both gases. As shown in Figure 2a, for *P. umbilicalis* Control (PU-CTRL), between day 0 and 15, the O_2_ concentration decreased by about 10% (half the initial value, ranging from 20.00% to 10.60%) and the CO_2_ concentration increased by a similar extent (around 9%, ranging from 0.20% to 9.13%). As shown in Figure 2b, for *U. lactuca* Control (UL-CTRL), the O_2_ concentration decreased by about 4% (from 20.00% to 15.83%) and the CO_2_ concentration increased to the same extent (from 0.20% to 4.50%) with no significant differences for O_2_ between days 3 and 15, and for CO_2_ between days 3 and 12. It is also worth mentioning that the CO_2_ concentration on day 15 for the UL-CTRL was not significantly different from days 0, 9, or 12.

### 3.2. Physicochemical Characterization

While none of the treatments seemed to be efficient in maintaining the original color of the seaweeds—a discoloration of both seaweeds was observed for all of them—the opposite seemed to be true for the texture, which was well preserved, especially with the MAP treatment.

#### 3.2.1. Color

**Figure 3:**
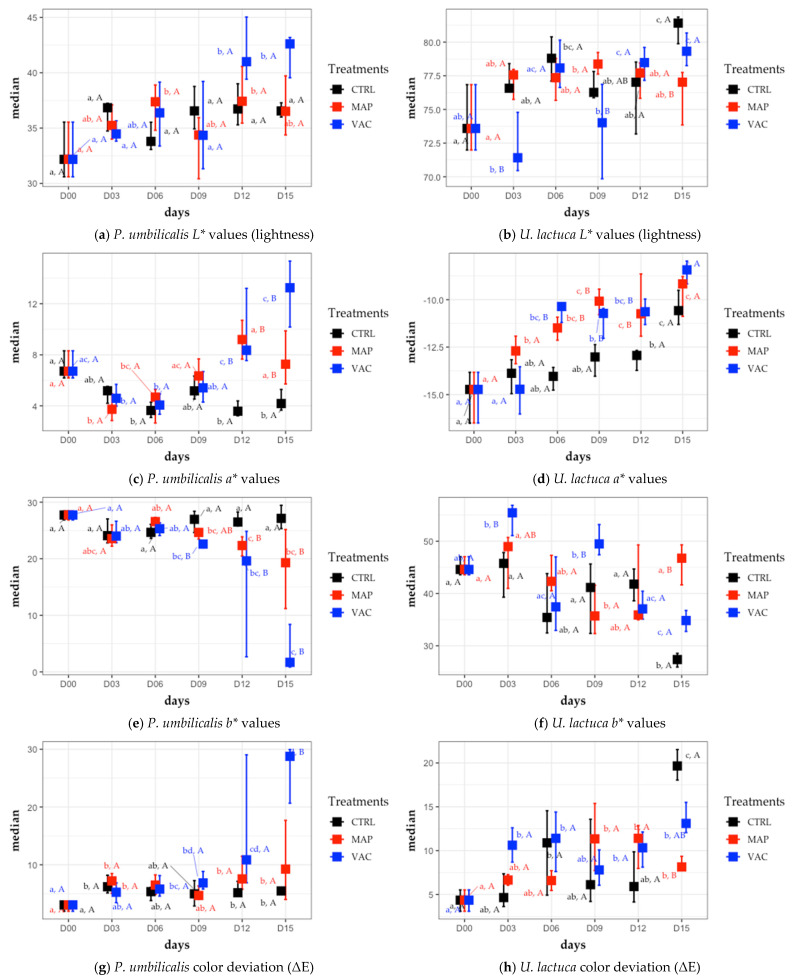
Color changes throughout storage days for *P. umbilicalis* and *U. lactuca* preserved by different treatments. The results are expressed as the CIELAB coordinates for L*, a*, b*, and the calculated color deviation (Δ*E*). Notes: Medians and interquartile range (IQR) from duodecuple determinations on each experiment. Medians followed by a different lowercase letter are significantly different (*p* < 0.05) throughout storage days. Medians followed by a different uppercase letter are significantly different (*p* < 0.05) among treatments.

As shown in Figure 3a,b, the coordinate L* became slightly lighter for both seaweeds throughout the storage days. Although no significant effect of the treatments was observed in *P. umbilicalis* (PU), the lightening seemed to be more pronounced on *U. lactuca* (UL) in the CTRL and VAC samples.

Regarding the a* coordinate (Figure 3c), a difference was observed for the MAP and VAC treatments for PU relative to CTRL on days 12 and 15. From day 9 onwards, the values seemed to increase for red, especially for the VAC treatment, which doubled (from 6.72 to 13.25). For UL (Figure 3d), whose coordinates were on the negative (green) side of the a* axis, the MAP and VAC treatments were statistically different compared to CTRL from day 6 onwards. The values for the CTRL, MAP and VAC treatments grew in different patterns and, on day 15, an increase was observed from −14.73 on day 0 to −10.58, −9.17, and −8.43, respectively.

For the b* coordinate of PU (Figure 3e), a difference relative to CTRL could be observed for the MAP and VAC treatments from days 12 and 9 onwards, respectively. While the values did not change significantly for CTRL throughout the storage days, the values for the MAP treatment ranged from 27.74 on day 0 to 19.31 on day 15. For the VAC treatment, a median of 1.68 was observed for the last day (6% of the initial value), which demonstrates an almost inversion from positive to negative values—i.e., a change from the yellow component to the blue one. For UL (Figure 3f), a large variation in the values in several directions was noticed, making it difficult to detect a trend. Nevertheless, on the last day, a decrease in the values for the CTRL and VAC treatments could be observed, while the MAP treatment seemed to maintain the reference value.

In other words, a positive change of the a* value on the red axis together with a negative change of the b* value on the yellow axis in the CIELAB color space indicated a shift in color towards a maroon (dark brownish red) color for PU. For the VAC treatment on day 15, it could also be stated that, in the case of overturn from yellow to blue, in combination with a redder tone with respect to the original color, a resulting violet color could be perceived. For UL, a positive change in the a* value on the green axis together with a negative change in the b* value on the yellow axis indicated a change in color towards a more olive-green tone (see Supplementary Material, Table S1).

The Δ*E* values for PU (Figure 3g) showed a larger color deviation for VAC, especially on days 12 and 15. CTRL showed the best results for PU on the last day of the experiment, but the color did not differ statistically from the MAP treatment. For UL (Figure 3h), major variations were observed in the values for CTRL, particularly on day 15. Therefore, the MAP treatment seemed to be the technique that best preserved the color of both seaweeds.

#### 3.2.2. Texture

**Figure 4:**
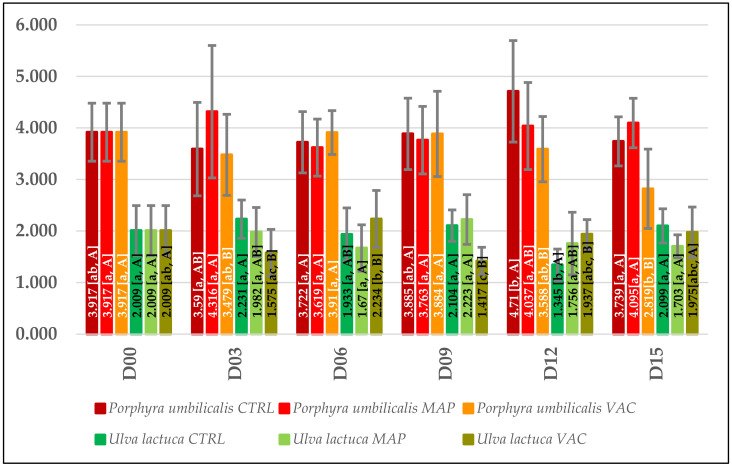
Texture (hardness) throughout storage days for *Porphyra umbilicalis* and *Ulva lactuca* preserved by different treatments. The results are expressed in newtons (N). Notes: Means ± SD from decuple determinations on each experiment. Means across columns of the same color followed by a different lowercase letter are significantly different (*p* < 0.05) throughout storage days. Means in the same group of bars for each storage day and seaweed followed by a different uppercase letter are significantly different (*p* < 0.05) among treatments.

As shown in Figure 4, no significant difference between the CTRL and MAP treatments was found for most days. The exception was the VAC treatment, which was significantly different from CRTL on days 12 and 15 for PU and on days 9 and 12 for UL.

For PU, the hardness values for the CTRL and MAP treatments were relatively stable throughout the storage period, while the VAC treatment showed a significant decrease in hardness on day 15 (from 3.917 N to 2.819 N). In the case of UL, the hardness values for the CTRL, MAP and VAC treatments were also stable during storage. On day 15, none of the samples were significantly different in terms of texture from CTRL on day 0.

### 3.3. Microbiological Analysis

**Figure 5:**
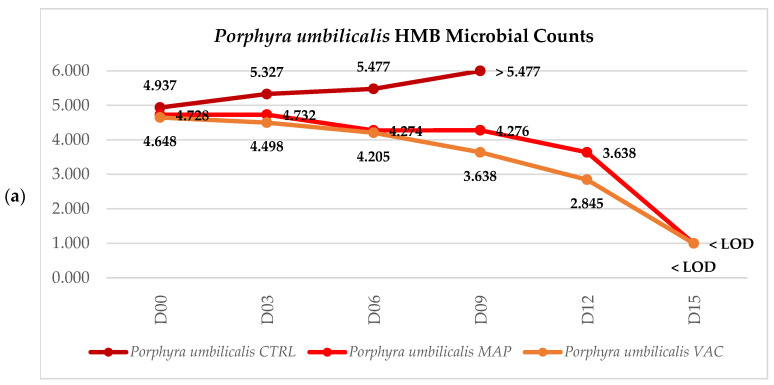

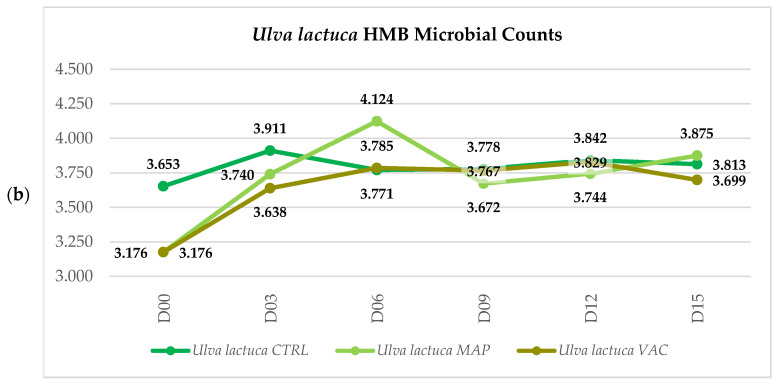
Microbial counts for heterotrophic marine bacteria throughout storage days for (**a**) *P. umbilicalis* and (**b**) *U. lactuca*, preserved by different treatments. The results are expressed as the logarithm of colony-forming unit per gram of sample (log CFU·g^−1^). Notes: Days 0, 3, 6, 9, 12, and 15 (n = 2 Petri dishes × 2 dilutions × 1 repetition = 4, for each treatment); LOQ: limit of quantification; LOQ < 1 log CFU·g^−1^ for heterotrophic marine bacteria.

The results revealed that the initial microbial counts of HMB for *P. umbilicalis* and *U. lactuca* were relatively high, ranging from 3.335 ± 0.275 log CFU·g^−1^ for UL to 4.775 ± 0.148 log CFU·g^−1^ for PU, as shown in Figure 5. Throughout the storage period, the microbial counts varied depending on the preservation treatment and the type of seaweed.

For PU (Figure 5a), the MAP and VAC treatments resulted in a decrease in the microbial count of HMB throughout the storage period, while the opposite occurred for CTRL. On day 6, the microbial count for CTRL achieved the value of 5.477 log CFU·g^−1^, which would make the seaweed unfit for consumption, and the values were even higher (above the limit of quantification) on day 9. From day 9 onwards, it was possible to notice a sharp drop in the microbial load for both the MAP and VAC treatments, particularly in the case of the VAC samples. On day 15, the microbial load for HMB was below the limit of detection for both the MAP and VAC treatments.

For UL-CTRL (Figure 5b), the HMB microbial counts were generally lower than those for PU. The VAC treatment showed the lowest microbial counts throughout the storage period, with values ranging from 3.176 log CFU·g^−1^ on day 0 to 3.699 log CFU·g^−1^ on day 15, while for the MAP treatment, the microbial counts showed the highest variation during the storage period (from 3.176 log CFU·g^−1^ on day 0 to 4.124 log CFU·g^−1^ on day 6). However, the microbial counts on day 15 were remarkably similar for CTRL and both treatments. Therefore, it can be inferred that the microbial counts for UL remained relatively constant across all treatments during the storage period.

The samples were also analyzed for pathogenic bacteria such as coliforms, *Escherichia coli*, coagulase-positive Staphylococci, and *Vibrio* spp. (quantification), *Salmonella* spp. and *Listeria monocytogenes* (identification). All analyses were below detection limits (<LOD) or not detected (N/D), except for coagulase-positive Staphylococci where non-characteristic black colonies without halos grew in the incubation medium of PU. These results suggested that the seaweeds collected were suitable for human consumption. For more details, see Supplementary Material Table S1.

### 3.4. Chemical Characterization

#### GC–TOF-MS Analysis

After harvesting and, especially, during the storage period, the seaweeds release VOCs which, concisely, can arise from three main sources: compounds derived from their metabolism, those from microbial activity, and substances present in seawater that adhere to their surface (including contaminants) [56,57,58]. The analysis described here allowed for the detection of 126 VOCs for *P. umbilicalis* and 148 for *U. lactuca*. These compounds could be divided into groups characterized by the presence of the following functional groups: alcohols, aldehydes, carboxylic acids, esters (of fatty acids and lactones), hydrocarbons, and ketones (including acyloins). A “diverse” group was used to aggregate minor VOC categories.

**Figure 6:**
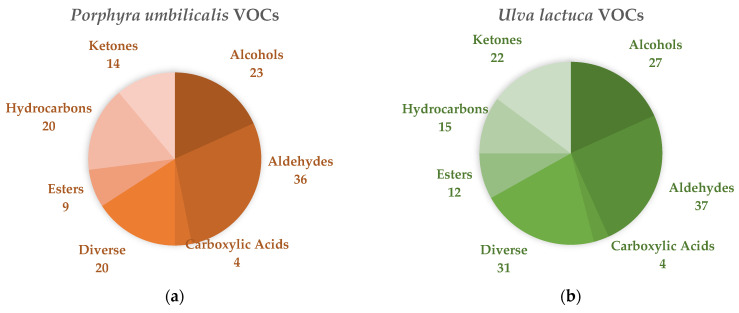
Functional group categories and number of VOCs identified by GC–TOF-MS for each: (**a**) *P. umbilicalis* and (**b**) *U. lactuca*.

As shown in Figure 6, most of the VOCs in both seaweeds belonged to the aldehyde group, of which 36 VOCs were identified for PU and 37 VOCs for UL. The next most represented category was that of the alcohols, of which 23 VOCs were identified for PU, and 27 VOCs in UL. The third most abundant group was hydrocarbons for PU (20 VOCs) and ketones for UL (22 VOCs). In the “diverse” group, the categories of compounds included were different for each of the seaweeds, as well as the number of compounds for each category. The following VOCs were identified for PU: three nitrogen-containing, one sulfur-containing, one epoxide, six furans, one indan, one ketose, two phenols, one pyridine, and four terpenoids. For UL, the following VOCs were identified: one nitrogen-containing, four sulfur-containing, four halogenated VOCs (two bromine-containing, one chlorine-containing, one iodine-containing), one benzothiazole, one epoxide, eight furans, three indans, one naphthol, four phenols, one terpene, and three terpenoids.

Regarding the characterization of the functional groups throughout the storage days, there was not a great variation in terms of the number of compounds identified for most samples, with a few exceptions (see Supplementary Material, Figure S1). For PU, there was an increase in aldehydes and compounds from the “diverse” group in VAC. The number of esters seemed to decrease in VAC, as well as the number of hydrocarbons, which was relatively smaller in relation to CTRL and MAP. For UL, in all samples, the number of alcohols (except for MAP on day 15 and VAC on day 6) and ketones (except for MAP on day 12 and VAC on day 6) appeared to increase in comparison with day 0, with greater intensity for those from CTRL. The esters seemed to decrease with storage time for all the treatments. It is important to notice that some acetic acid was found in VAC throughout storage, whereas it was not detected in CTRL or MAP—except on day 3, where a trace amount of this compound was detected in the latter.

Tukey HSD results (see Supplementary Material, Table S2) for *P. umbilicalis* revealed that there were no significant differences among both the MAP and VAC treatments and CTRL—only on day 6 did the medians differ in relation to day 0, despite two-way aligned rank transformation ANOVA showing only marginal significance for the days (Pr(>F) = 0.096001). The results for *U. lactuca* showed no difference between the MAP and VAC treatments, although both differed significantly from CTRL. While CTRL did not differ significantly between the days of storage, the MAP and VAC treatments showed significant differences in relation to day 0; nevertheless, no significant changes were observed from day 3 onward.

For PU, the first principal component (PC-1) accounted for 50% of the variance of the samples, while the second principal component (PC-2) contributed 35%, together explaining 85% of the total variance. For UL, the PC-1 accounted for 43% of the variance of the samples, the PC-2 contributed 27%, and the third principal component (PC-3) explained 12%, together describing 82% of the total variance.

**Figure 7:**
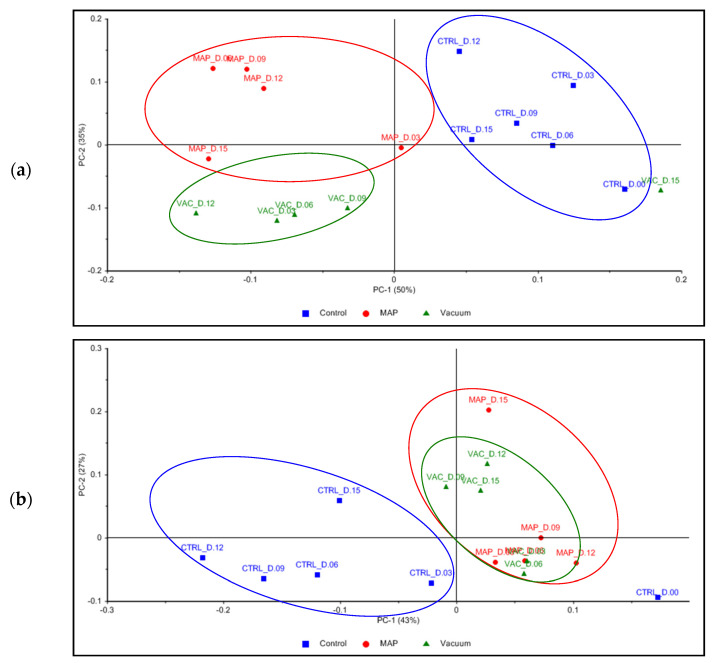
Principal component analysis (PCA) of the seaweed treatments and storage periods: Score plot graphics of PC-1 vs. PC-2 for (**a**) *Porphyra umbilicalis* and (**b**) *Ulva lactuca*.

Examining Figure 7a, the PCA was able to group the PU samples by type of treatment, which were in different zones of the score plot—except for the VAC treatment on day 15. While PC-1 was capable of separating CTRL, mainly in the 1st quadrant, from the treatment’s clusters, PC-2 allowed for the separation of the VAC and MAP treatments mainly in the 3rd and 4th quadrants, respectively. Regarding the UL samples (Figure 7b), the PCA was not able to discriminate between the MAP and VAC treatments, both being located close to the origin and mostly on the positive axis of the PC-1, while the CTRL samples were located on the negative axis of the PC-1—except for CTRL on day 0, which was positively associated with the underlying patterns captured by the PC-1.

**Figure 8:**
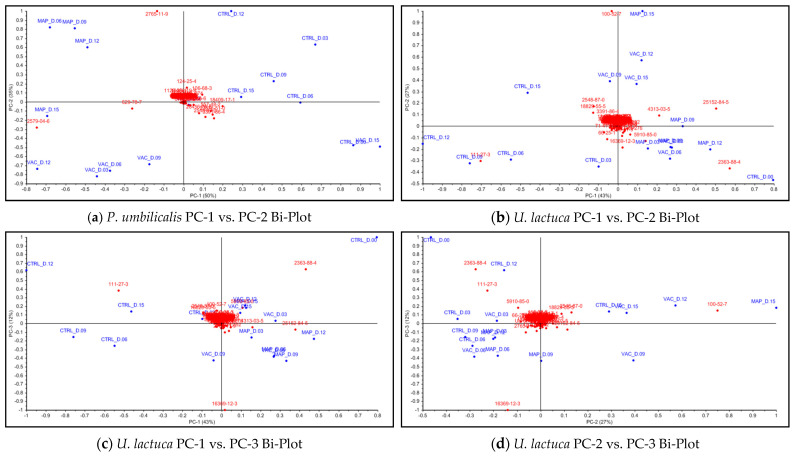
PCA of the seaweed treatments and storage periods. Bi-plot graphics containing scores and loads of: (**a**) PC-1 vs. PC-2 for *P. umbilicalis*, (**b**) PC-1 vs. PC-2 for *U. lactuca*, (**c**) PC-1 vs. PC-3 for *U. lactuca*, and (**d**) PC-2 vs. PC-3 for *U. lactuca*.

As shown by Figure 8a, the compounds “8-heptadecene (isomer 2)” (code 2579-04-6) and “heptadecane” (code 629-78-7) in the 3rd quadrant and “pentadecanal” (code 2765-11-9)” in the 4th quadrant seemed to be the most important variables to explain the changes in PU samples throughout storage days.

Examining Figure 8b–d, it can be seen that the compounds “2,4-decadienal, (*E,E*)-” (code 25152-84-5) in the 1st quadrant, “2,4-decadienal (isomer)” (code 2363-88-4) in the 2nd quadrant, “1-hexanol” (code 111-27-3) in the 3rd quadrant, and “benzaldehyde” (code 100-52-7) in the 4th quadrant of PC-1 vs. PC-2 seemed to be the most relevant variables that accounted for the variance in UL samples. Analyzing PC-2 vs. PC-3., “8-heptadecene (isomer 1)” (code 16369-12-3) in the 3rd quadrant was distinguished as another relevant variable.

In the case of PU seaweed (Table 1), the concentration of 8-heptadecene (isomer 2) appeared to increase over the storage period for both the CTRL and MAP treatments. By day 15, the MAP treatment exhibited a concentration approximately twice that of CTRL, with values of 27.679% and 14.502%, respectively. For the VAC treatment, the concentration on day 15 showed the lowest recorded level of 5.527%. Heptadecane displayed a similar behavior; by day 15, on one side, the concentration of heptadecane in MAP was approximately five times that of CTRL (with values of 10.283% and 2.037%, respectively), while, on the other side, the lowest level of 1.143% was recorded for the VAC treatment. Regarding pentadecanal, there was a significant increase in concentration for both CTRL and MAP on day 3, with values of 17.544% and 8.747%, respectively, compared to the initial concentration of 1.826%. On day 6, pentadecanal decreased in CTRL while increasing in MAP, showing a little oscillation on day 9 and, on days 12 and 15, the concentrations were very close. For the VAC treatment, the concentration initially decreased but remained relatively stable during storage, averaging around 1.755%.

In the case of UL seaweed (Table 2), both (*E,E*)-2,4-decadienal and its isomer exhibited similar behavior in CTRL over the storage period. Initially, they contributed a major share of volatiles on day 0 (12.425% and 27.565%, respectively) and, to a lesser extent, on day 3 (4.018% and 9.401%, respectively). However, the values kept falling from day 6 onwards. Regarding the MAP treatment, after an initial drop on day 3, the concentrations of 2,4-decadienals kept increasing for both compounds until day 12, with more intensity for (*E,E*)-2,4-decadienal. By the end of the storage period, there was another accentuated decrease. Regarding the VAC treatment, the values of both 2,4-decadienals also presented some variation, although they displayed a similar behavior: the values kept falling, with more intensity for the isomer, reaching the lowest concentration on day 9. On day 12, they increased again, with minor changes on day 15.

In the case of 1-hexanol, its concentration increased in CTRL until day 12, with values ranging from 0.133% to 24.474%, after which it decreased to the value of 4.760%. In the other treatments, their values showed a slight rise compared to the control on day 0 but remained relatively stable until the end of the storage period. Regarding benzaldehyde, it experienced a considerable increase in concentration for CTRL and both MAP and VAC treatments, with a notable importance of samples packed under a modified atmosphere (from 2.287% to 33.800%). As for 8-heptadecene (isomer 1), it demonstrated a similar behavior across all samples. There was a tendency for its concentration to increase until day 9 and to subsequently decrease, albeit remaining relatively above the initial value of 6.000%.

## 4. Discussion

Common challenges faced by minimally processed foods include the occurrence of decay and/or senescence, emergence of off-odors, discoloration, and softening of the tissue [60]. These major problems related to food processing and storage will be addressed in the following subsections for the studied seaweed species.

### 4.1. Composition of Headspace Gases

Most seaweeds do not undergo photorespiration (i.e., respiration in the presence of light), unless in special circumstances—i.e., high O_2_ concentration in combination with low CO_2_ levels, during reproduction, tissue repair or growth, and in response to a change in the pH of the medium. If photorespiration is negligible in the case of seaweeds, then the same does not happen with respiration in dark conditions, which is related to biosynthesis, carbon balance, and the maintenance of cellular health and functions related to photosynthesis [61].

The results obtained indicate that the MAP treatment and storage conditions were able to keep photosynthesis and respiration rates low, once the O_2_ levels were maintained close to 0% and the CO_2_ decreased slowly throughout the storage days. It should be considered that a small part of the CO_2_ dissolves in water and that, if necessary, carbon uptake by seaweeds comes mostly from the bicarbonate ions (HCO_3_^−^) through a reaction catalyzed by the surface-bound enzyme carbonic anhydrase [62]. The low permeability of the PA/PE polymers used to preserve the samples should also be mentioned because they likely played a role in effectively reducing gas exchange with the external environment [63]. Argon may similarly have contributed due to its ability to disrupt the binding of oxygen to enzymatic receptor sites [64].

Regarding the controls, an increase in CO_2_ concentration in the headspace followed by a similar decrease in O_2_ was observed. This occurred at variable rates for PU and UL, with greater activity being observed in the former. The ratio for CO_2_ release and O_2_ consumption was close to 1 in both seaweeds, which is consistent with the average respiration quotient (RQ) for seaweeds [65]. However, it was expected that the O_2_ produced by photosynthesis could compensate, by some orders of magnitude, its consumption by respiration (also known as “net photosynthesis”) [66]. The results suggested that most likely there was no significant photosynthetic activity due to the low content of HCO_3_^−^ or nutrients in the medium (or CO_2_ in the headspace of VAC samples) since the packages were sealed without seawater [61,67,68]. For seaweeds to achieve balanced growth, they must obtain nutrients in the correct proportions [69,70]. Otherwise, the carbon fixed through photosynthesis cannot be effectively utilized to produce new biomass, instead being excreted as dissolved organic carbon or stored as polysaccharides [71]. Another hypothesis is based on the fact that, under abiotic stress, seaweeds are prone to produce “reactive oxygen species” (ROS) that can damage chlorophylls in the chloroplast, thus reducing photosynthetic activity [72,73,74]. Therefore, it can be assumed that the change in the composition of headspace gases was mainly due to dark respiration (maybe photorespiration induced by stress or reproduction) combined with photosynthesis suppression [75].

### 4.2. Physicochemical Characterization

#### 4.2.1. Color

Lee et al. [76] investigated the effect of supercooling conditions on the conservation of *Pyropia yezoensis*. This was the only study encountered on the effect of storage conditions on the color of fresh “laver” seaweed. The main remarks were that L*, a* and b* values of the refrigerated fresh samples (stored at 5 °C) were affected by the storage temperature with the CIELAB values increasing more than that of the other samples stored at constant and step-cooling (−2 °C) or frozen (−18 °C), during the storage period of 15 days. This is not in accordance with our results in which there was a small decrease in a* and no significant difference in b* for PU-CTRL after 15 days of storage. It is possible that the differences between the results obtained by Lee et al. and our results for PU-CTRL were due to the different experimental settings such as illumination [77,78]. Because of the differences in how the MAP and VAC samples were treated, it is not reasonable to establish correlations between the studies.

Olmo et al. [32] did not find significant differences for L* in *Ulva lactuca* in the first 30 days of storage for untreated samples (controls) refrigerated at 4 °C. No significant difference was found for parameters a* or b* for the control; however, similarly to our study, a tendency towards an increase in a* on the green side of the axis and a decrease in b* on the yellow side of the axis might be observed. According to the authors, the main changes in color parameters for the control could be attributed, in part, to the amplification of antenna complex pigments under the low-light conditions. Pinheiro et al. [20] evaluated the impact of storage time in the CIELAB coordinates for *Ulva rigida* treated under different conditions (air-dried, brined, salted with 28% NaCl and salted 40% with NaCl). Despite the differences in the experimental conditions, the conclusions were like those observed in the present study for the L* and a* coordinates—i.e., there was a tendency for both values to increase, with a* being on the green side of the axis. Sánchez-García et al. [79] studied the impact of the storage period (12 days) and two different temperatures (4 °C and 16 °C) in the color profile of untreated *Ulva rigida*. The results were in accordance with our findings: an increase in a* on the green part of the axis, a decrease in b* on the yellow part of the axis and ambiguous values for L* that decreased in samples stored at 4 °C and increased in those at 16 °C. The key findings were that temperature appeared to have a significant impact on color, especially when *U. rigida* was stored at higher temperatures. Furthermore, from the sixth day of storage onwards, a more pronounced browning of the seaweed was observed. Browning in seaweeds is related to the destruction of the tissue itself, breakdown of chlorophyll compounds (into pheophytin and pheophorbide), alteration caused by enzymatic (e.g., polyphenol oxidase) and non-enzymatic activity, as well due to microbial growth [79,80].

Different from the treatments that did not seem to significantly affect lightness in PU and only affected UL-VAC on day 6 and UL-MAP on day 15, our results showed that storage time had a positive impact on L* values (whitening) for PU-VAC, UL-CTRL and UL-VAC. Harrysson et al. [81] studied the color of *Porphyra umbilicalis* and *Ulva fenestrata* after oven-drying at 40 °C, and during subsequent storage for ≥370 days under light, semi-light and dark conditions. The main conclusion regarding lighting conditions and storage time was that the more intense the light used and the duration of the storing, the greater the increase in L* values. Moreover, the available evidence suggests that an elevation in L* values may occur due to pigment breakdown as a result of light oxidation, auto-oxidation (mainly, because of the presence of ROS), and even enzymatic reactions [26,81,82,83].

The changes in the a* and b* coordinates for PU and UL were both affected by treatments and storage time. For PU, a small decrease was observed followed by an increase in a* values, reflected in changes in red tones, for the MAP and VAC treatments, probably due to changes in phycobiliproteins, in particular phycoerythrins that have a reddish color [77,78]. It should be noted that the increase observed from day 9 onwards seemed to be due to a red exudate (phycoerythrin release) observed mainly in PU-VAC, in a similar way to what was witnessed in the alga *Gracilaria salicornia* (Rhodophyta) preserved under vacuum [35]. Chlorophylls appeared to play a minor role in changing a* values for “laver” seaweeds as a result of their low concentration in these species [84]. With regard to b*, the significant decrease for both treatments (with more intensity for PU-VAC) seemed to occur due to alterations in the composition of carotenoids and phycocyanin-type phycobilins (of bluish coloration) [81,85]. Phycocyanins were more stable than carotenoids, and the phycocyanin/carotenoids ratio appeared to increase with time, promoting a decrease in yellowish tones and an increase in bluish tones in red seaweeds [85].

**Figure 9:**
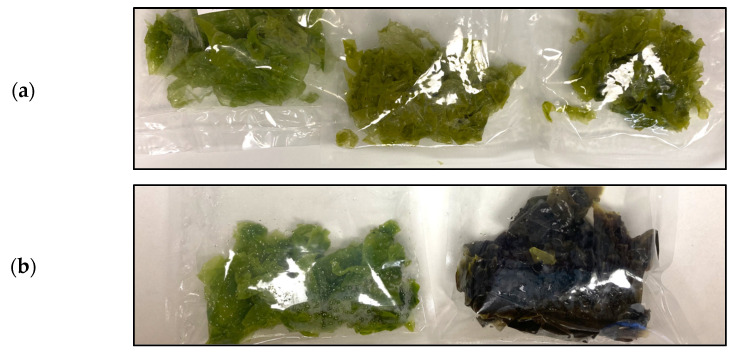
(**a**) Color differences for *U. lactuca* between treatments on *day 9*: *control* (left), *MAP* (center), and *vacuum* (right); (**b**) Color of the seaweeds *U. lactuca* (left) and *P. umbilicalis* (right) on *day 0*.

For UL, all treatments implied an increase in a* (less green intensity) over the storage time, which possibly occurs due to the degradation or adaptation of the chlorophylls to the experimental conditions [20,32,79]. Like PU, b* values for UL also decreased with time, except for UL-MAP, which was not significantly different from day 0. These variations in b* values may have been exclusively caused by changes in the carotenoid composition [20,79]. Carotenoid content in seaweeds is directly affected by ROS through quenching activity, and indirectly by inducing the carotenoid biosynthetic pathway [86]. As both the VAC and MAP treatments involved the elimination of oxygen, it is possible that the absence of O_2_, together with the presence of Ar and high CO_2_ in the MAP samples, helped in the maintenance of carotenoids by suppressing oxidation reactions and/or enzymatic activities [86,87]. A more yellowish coloration could be observed with the naked eye in samples processed by the MAP and VAC treatments on day 9 relative to CTRL (see Figure 9a).

The adaptation or degradation of pigments due to light, extended duration and other storage conditions, together with metabolic responses to diverse sources of stress such as starvation (nutrient depletion) and cold damage, are the most possible hypotheses for the increasing L* values and the changes observed in a* and b* for both PU and UL seaweeds [20,32,79,88,89,90,91]. It should also be taken into consideration that some of the variations observed throughout the storage days can arise from the heterogeneity of the samples, as can be observed in the different shades of green for UL and brown with discolored edges for PU, both presented in Figure 9b.

#### 4.2.2. Texture

The studies encountered in the literature about the texture analysis of fresh seaweeds subjected to different storage conditions or treatments are scarce, and the methodologies used vary greatly between trials. It was not possible to find analyses for fresh *P. umbilicalis* (or any other laver seaweed) over a storage period. Therefore, the results for other red seaweeds or those with laminar thalli were used as references.

The Nayyar & Skonberg [92] texture profile analysis (TPA) for *Palmaria palmata* (Rhodophyta) showed that hardness values were notably impacted by both time (2 weeks) and temperature (2 °C and 7 °C), with values decreasing gradually over time and more intensively for higher storage temperatures. For *Gracilaria salicornia*, the majority of the thalli had lost their firmness by the end of the storage period of 6 days at 16 °C or 21 °C in the light or dark. However, this decline could not be correlated with either the storage temperature or light conditions due to the considerable variability in the texture values [35]. These results are not in accordance with our findings for PU-CTRL, although it must be considered that the *P. umbilicalis* has different morphology and textural characteristics from *P. palmata* and *G. salicornia*.

Research on the use of HPP for *U. lactuca* did not find significant texture differences for the control on the 30th day of storage at 4 °C, just as with UL-CTRL in our research. This was not true for the same seaweed treated with 400 MPa, which seemed to lose hardness only after 30 days of storage. Cell disruption and lyase activity (bacterial or endogenous) seemed to be the reason for such deterioration [32]. TPA on the sensory characteristics of *U. rigida* indicated that the hardness values were influenced by storage duration (12 days) and temperature (4 °C and 16 °C), with a gradual decrease observed over time at both temperatures. This weakening of the cell structure may have occurred due to chemical and enzymatic reactions, and even microbial activity, making the tissue more fragile and susceptible to breakage [79].

Based on what was previously discussed, it appears that seaweeds can present different behaviors depending on the evaluated species or experimental conditions [32,79,92]. Furthermore, the same species may show high variability in their textural properties related to the collection site, uneven decay, or tissues analyzed [35,42,43]. These reasons account for the varying behavioral patterns observed in the addressed works.

Our results showed that hardness values for the CTRL and MAP treatments were generally comparable, whereas the VAC treatment exhibited some degree of variability, with *P. umbilicalis* prone to become less resistant on day 15. While many studies suggest that the decline in hardness of the seaweeds may be attributed to chemical or biological factors, there is no evidence in the current study to support the deterioration of seaweed by microorganisms, as PU-VAC exhibited the lowest microbial count on day 15. Most likely the variations in VAC treatment resulted from the compression of the seaweeds by the vacuum package that may have caused some random break in the cell matrix in a similar way to what happened for the HPP-treated seaweeds. The breakdown of cell structure and aging, alongside a loss of intracellular content, could have promoted autolysis reactions in samples [32]. The key finding was that MAP may be a more effective preservation method in maintaining the textural properties of fresh seaweeds compared to VAC.

### 4.3. Microbiological Analysis

Some of the reviewed studies refer to a negligible presence of yeast and molds in fresh seaweeds [93,94,95]. Similarly, halophilic lactic acid bacteria present in seaweeds do not seem to grow in culture media [96]. Additionally, the microbial load in Marine Agar (heterotrophic marine bacteria) consistently exceeds that of Plate Count Agar, which assesses the total or viable bacterial growth in a sample [32,33]. Consequently, the focus is placed on evaluating the primary pathogenic agents, at the beginning and end of the test, and determining whether the total microbial load of marine microorganisms throughout the storage time complied with the regulatory parameters outlined by local guidelines in the European Union (EU) (see Supplementary Material, Table S3).

For PU, there was a decline in heterotrophic marine bacteria (HMB) over time in the MAP- and VAC-treated seaweeds, whereas in CTRL, the CFUs continued to increase until they surpassed the quantification limit. The treatments appeared to have affected aerobic bacteria, as they continued to grow in the control group, which had approximately 20% oxygen [97]. It is noteworthy that many of the bacteria that inhabit the surface of red marine macroalgae are obligate or facultative aerobe, such as the common *Bacillus* spp. and *Virgibacillus* spp. [98,99]. Numerous seaweeds release secondary metabolites with antimicrobial and antifouling activities—i.e., able to regulate bacterial growth and prevent or inhibit the attachment of unwanted organisms on their surface [93,100,101]. This is particularly valid for red seaweeds that, under certain circumstances, can release a variety of compounds with antibiotic activity against Gram-positive and -negative bacteria [102]. Therefore, the treatments must have reduced the total aerobic bacterial load and possibly triggered some metabolic activity in PU with the release of secondary metabolites that affected the microbial population.

Lee et al. [76] in their study emphasized the importance of low-temperature storage in minimizing microbial growth. Notably, the authors utilized sterilized Plate Count Agar instead of Marine Agar to assess the Total Aerobic Count (TAC). Their results showed that the initial TAC for fresh *Pyropia yezoensis* samples was 3.28 log CFU·g^−1^. After 6 days of storage, the TAC for refrigerated samples rose to 4.25 log CFU·g^−1^, which was significantly higher compared to samples stored under other conditions such as freezing and supercooling. This study also showed significant growth for the control of *P. yezoensis* during the storage period, whereas the other treatments exhibited a consistent microbial load, with a slight decrease observed from day 6 onwards. These results are consistent with our findings.

For UL, the microbial load remained relatively stable in all treatments throughout the storage days, and neither the MAP nor VAC treatment seemed to have had any significant effect on lowering microbial counts. Liot et al. [94] studied the microbiology of *Ulva lactuca* and *Palmaria palmata* submitted to different washing procedures and stored at 4 °C for 14 days. The authors noticed that mesophilic aerobes maintained relatively stable levels throughout the storage days, particularly in non-washed and seawater-washed samples of *U. rigida* and *P. palmata*, for which the initial microbial population ranged from 10^3^ to 10^5^ CFU·g^−1^, in a similar way to what happened in our study to UL.

Olmo et al. [100] in a study on the microbial diversity of the seaweeds, observed a decrease in bacterial diversity at the genus and species level for all the seaweeds at the end of the storage period [100]. In another study from Olmo et al. [99], on the bacterial diversity in dried seaweeds (including *U. lactuca* and *P. umbilicalis*), a lower bacterial diversity was observed compared to fresh marine macroalgae (specifically, for the case of *U. lactuca*), with a prevalence of bacteria from the Bacillaceae family for both seaweeds. Despite the study being conducted on dried samples, it can be deduced that *U. lactuca* likely possesses a higher bacterial diversity compared to *P. umbilicalis*. Due to the constraints imposed by traditional culture-based techniques, in comparison to molecular methods (e.g., 16S rRNA gene sequencing), it was not possible to determine the diversity of the microflora regarding the biological classification [103]. Thus, despite the microbial load remaining relatively stable, it was not possible to know to what extent there were variations at the taxonomic level.

To conclude, the anaerobic conditions of both the MAP and VAC treatments, together with the bactericidal effect of the secondary metabolites released by seaweed, appeared to account for the reduction in the overall microbial load, below the limits of detection, observed in the PU samples. Although the microbial load in UL remained steady for CTRL and both the MAP and VAC treatments, there is a possibility that there were modifications in the bacterial composition of the samples. At the end of the storage period, both seaweeds treated with MAP and VAC met the primary microbiological criteria outlined in the legislation: with a particular emphasis on PU, in which the treatments yielded notable improvements, while for UL neither treatment seemed to have a significant effect on reducing microbial load. As a result, these treatments guaranteed the consumption of the studied seaweeds for a minimum period of 15 days in terms of food safety.

### 4.4. Chemical Characterization

#### GC–TOF-MS Analysis

Seaweeds are known for their unique sensory properties, and certain terms are commonly used to describe their distinct flavors and aromas. Some descriptions frequently associated with “laver” (*Porphyra* spp., *Pyropia* spp., and *Neopyropia* spp.; Rhodophyta) are a distinct sea taste, evoking the essence of the ocean when fresh; the dried form exhibits flavors similar to mushrooms and dehydrated fruits such as raisins; and, when toasted or cooked, it takes on a more fishy profile, suggestive of roasted sardines. For “sea-lettuce” (*Ulva* spp.), the most commonly used organoleptic descriptors are: when fresh, slightly bitter with green and fatty aromas and some notes of green vegetables such as cucumber and herbs (due to the presence of various aldehydes); when dried, the aroma resembles the marine environment, due to the production of dimethyl sulfide (DMS), with notes of cut grass or *matcha* (green tea) [26,104,105].

López-Pérez et al. [106] identified in *P. umbilicalis* the highest levels of total volatile alcohols and ketones in contrast to the other six species of dehydrated seaweed they studied, including *U. lactuca*, while no halogenated compounds were detected. Consistently, the aroma of “laver” seaweed appears to be primarily influenced by the presence of alcohols and ketones, with a lesser contribution from aldehydes and sulfur compounds (see Supplementary Material, Figure S2). Despite being abundant, hydrocarbons do not seem to significantly contribute to its aroma, and the commonly found halogenated compounds in various red marine macroalgae species do not thrive in this particular seaweed (except for trichloroethylene) [26,107,108]. These results are consistent with our findings. Regarding UL, aldehydes not only prevail as the most abundant VOCs in terms of diversity, but also exhibit the highest concentration in relation to the chromatogram area (see Supplementary Material, Figure S3). These findings align with the description provided by Fujimura & Kawai [26] regarding the distinctive flavor constituents of *Ulva australis* (formerly *Ulva pertusa*).

PU showed a high concentration of 8-heptadecene (isomer 2) and heptadecane, which apparently do not influence the aroma of the samples due to their high odor thresholds [59]. The presence of these hydrocarbons may result from responses to treatments and biological activity of the marine macroalgae and associated microorganisms [26,108]. In seaweed, 8-heptadecene is involved in a protective mechanism against mechanical damage, functioning as a chemoreception molecule and biological pheromone during the wound healing process. Some seaweeds possess an enzymatic system capable of catalyzing eicosapentaenoic acid to produce this alkene [109]. The higher occurrence of 8-heptadecene in the VAC samples appeared to be related to the damage caused by the compression of the UL during the packaging process. Numerous studies have reported the presence of heptadecane or heptadecene in “laver” seaweeds [106,110,111], including heptadecane in *P. umbilicalis* [112] and 8-heptadecene in *Pyropia* spp. [109,113]. According to Kamenarska et al. [114], heptadecane and heptadecene were the predominant hydrocarbons found in red seaweeds collected from the Black Sea.

Pentadecanal appeared to have a relevant effect on the characterization of the aromatic profile of both the CTRL and MAP-treated PU due to its high abundance and lower odor thresholds (0.715 mg/kg). Pentadecanal exhibits a refreshing, wax-like scent with hints of floral notes [115,116]. Pentadecanal formation in seaweed seems to be related to α-oxidation of palmitic acid (PA) by lipoxygenases [117]. For some yet unknown cause, it seemed that the VAC treatment exhibited greater efficacy in inhibiting pentadecanal production.

For UL, (*E,E*)-2,4-decadienal and one of its isomers were the compounds with the highest concentration, particularly in the control on day 0. 2,4-decadienal is known for its greasy aroma, accompanied by green, fresh, and citrus notes. When present in high quantities, its flavor resembles that of fried chicken, with an underlying rancid aftertaste [115]. Given the low detection threshold (approximately 0.0003–0.00013 mg/kg) and high initial concentration of the 2,4-decadienal isomers, it can be inferred that these compounds significantly contributed to the characterization of the UL odor. In *Ulva conglobata*, it was demonstrated that 2,4-decadienals were produced via hydroxy eicosatetraenoic acids (HETEs), derived from arachidonic acid, by means of a crude enzyme that was isolated from the seaweed [118]. It is recognized that microbial activity can diminish the presence of these compounds, associated with a “fishy aroma” in fermented seaweeds [112,119]. Similarly, exposure to light also appears to contribute to their decline [120]. The significant decrease in 2,4-decadienals in CTRL seemed to be primarily influenced by the presence of microorganisms, rather than the level of light exposure, which was similar across all the samples.

1-Hexanol carries a distinctive green and herbaceous aroma, with a slight alcoholic hint, reminiscent of the scent of freshly cut grass [115]. Despite its medium odor threshold (1.21 mg/kg), it was reported that odor contribution in the seaweed *Ulva australis* was low [59,116]. 1-Hexanol can be generated through the peroxidation of unsaturated fatty acids. Additionally, aldehydes can be converted to their corresponding alcohols through a reaction catalyzed by aldehyde dehydrogenases (ALDHs) [121]. In the case of fermented seaweeds, it has been observed that 1-hexanal undergoes a transformation into 1-hexanol through the action of enzymes released by microorganisms [122]. In UL-CTRL, 1-hexanol reached its maximum at day 12, surpassing other abundant compounds such as 2,4-decadienals and 8-heptadecene (isomer 1), while in UL treated with MAP and VAC, its presence remained relatively low. Therefore, its occurrence appeared to be related to the aerobic activity of the microbiome found within the seaweed.

Benzaldehyde exhibits a sweet and fruity aroma, with a very characteristic flavor that resembles bitter almonds and cherries [115]. Although this compound possesses a moderate odor threshold (0.55 mg/kg) and seems to contribute to the aroma of certain species of *Ulva* spp., its influence is less noticeable compared to other aldehydes present [59,116]. In our study, benzaldehyde seemed to have an impact on the aroma profile of UL processed, particularly with MAP, although this influence appeared to be beneficial, considering the organoleptic attributes associated with the compound. There are many hypotheses about the metabolic pathway for the production of benzaldehyde in marine macroalgae, such as: thermally generated and derived from Strecker degradation of amino acids [123]; from aromatic compounds through enzymatic peroxidation by haloperoxidases, in the absence of halides [124]; and from amino acid derivatives by chemical routes as a consequence of lipid oxidation (this can occur either directly through free radicals derived from lipid hydroperoxides, or indirectly through reactive carbonyls derived from lipids) [125]. Similar to our results, López-Pérez et al. [56] identified benzaldehyde as the most abundant aromatic compound in *U. lactuca*. Its concentration seemed to be influenced by the storage time for all treatments, with an emphasis on samples preserved by MAP.

No existing literature was found regarding the characteristic aroma of 8-heptadecene, and its presence did not seem to impact the aroma of seaweeds from the genus *Ulva* spp. Some studies indicated 8-heptadecene as the primary hydrocarbon in marine green macroalgae [106,112,126]. The compound was isolated from the seaweed *Bryopsis maxima* (Chlorophyta), being identified as the (*Z*)-8-heptadecene stereoisomer [127].

Limited research exists regarding the changes in VOCs in seaweeds as influenced by storage duration. Stévant et al. [128] conducted a study to examine the impact of dry (6% moisture) and semi-dry (20% moisture) storage on the red seaweed *Palmaria palmata* for 126 days. Similar to the observations for PU, heptadecane initially decreased until approximately halfway through the storage period, after which it exhibited an upward trend, whereas both (*E,E*)-2,4-heptadienal and (*E,Z*)-2,6-nonadienal appeared to increase in the processed seaweeds. Furthermore, 1-octen-3-ol, a significant component in PU characterization, demonstrated an increase over the storage period, which in our case was only observed for PU stored under vacuum conditions.

In a study conducted by Sánchez-García et al. [79], aiming to investigate the changes in VOCs in *Ulva rigida* during a 12-day storage period at 4 °C or 16 °C, it was observed that 1-octen-3-ol increased throughout the storage period, while 2,4-heptadienal initially exhibited an increase but subsequently showed a decreasing trend towards the end of the storage period, which is also consistent with our findings. In contrast to our study, where benzaldehyde, hexanal and 2-heptenal exhibited an increase, the research indicated that benzaldehyde, hexanal and 2-heptenal decreased during the storage period. However, in our case, hexanal showed some oscillation, with a decrease observed only in the MAP treatment. Furthermore, relevant compounds, such as 2,4-decadienal or 8-heptadecene, were absent in their characterization of *U. rigida*. The differences in the findings between our research and Sánchez-García et al. can be attributed to the fact that the seaweeds in the above-mentioned study were subjected to freeze-drying during sampling, which may have promoted the loss of important VOCs.

Although there were some variations in VOCs, it is important to emphasize that no significant alteration in the distinctive aroma of the seaweed appeared to have occurred. Mirzayeva et al. [129], in their study regarding the characterization and differentiation of seaweeds based on their volatile composition, concluded that changes in VOCs tend to be more pronounced in relation to factors such as collection site, seasonal variations, and species differences, rather than changes resulting from pretreatments (preservation techniques). Furthermore, VOCs normally associated with the “fishy” aroma, which often leads to sample rejection by evaluation panels, seemed to decrease with storage time [130]. Additionally, compounds associated with pleasant aromas (e.g., benzaldehyde and pentadecanal) increased. These changes can be interpreted as being indicative of an improvement in the organoleptic properties of the treated seaweeds.

To confirm the key odorous components that contribute to the typical aroma of *P. umbilicalis* and *U. lactuca*, additional research is needed, employing techniques such as gas chromatography coupled with olfactometry (GC–O) and sensory analysis. These methods would help in screening and determining the essential compounds responsible for the distinctive fragrances associated with these seaweed species, as well the remarkable changes throughout the storage period.

The results allowed for the conclusion that both the MAP and VAC treatments appeared to be more efficient in maintaining the organoleptic properties of UL compared to PU, with no significant differences throughout the storage days. Despite the wide variety of VOCs identified by GC–TOF-MS, the PCA demonstrated that a few compounds could explain most of the variation in the samples of both seaweeds. Some of these compounds did not necessarily have any relevant impact on the sensory properties of PU and UL, and their generation was related to wound-healing processes and to the microbiome present in the seaweeds.

## 5. Conclusions

The results obtained on the impact of minimal processing on the seaweeds “laver” (*Porphyra umbilicalis*) and “sea-lettuce” (*Ulva lactuca*) showed that these allowed for safe consumption for a minimum period of 15 days. The composition of headspace gases indicated that the MAP treatment and storage conditions were able to keep photosynthesis and respiration rates low. The degradation of pigments caused discoloration in both seaweeds. However, the MAP preservation technique appeared to be the most effective in maintaining the color of both seaweeds. Regarding texture, our findings indicated that MAP may be superior to VAC in preserving the fresh seaweed’s textural properties. By the end of the storage period, both seaweeds treated with MAP and VAC complied with the primary microbiological criteria specified by regulations. Notably, both these treatments yielded significant improvements for PU, with microbial counts below the limits of detection on day 15, while for UL, neither treatment seemed to have a substantial effect on reducing the microbial load. Despite some variations in VOCs, there was no notable change in the distinct aroma of the seaweeds studied. However, both the MAP and VAC treatments demonstrated greater effectiveness in preserving the sensory characteristics of UL compared to PU, with no significant differences observed during storage.

Metabolic responses to diverse sources of abiotic stress such as temperature (cold damage), light and other parameters related to storage conditions (e.g., lack of nutrients or wounds caused by handling and packaging), together with metabolic activities at the level of the microbiome appeared to have accounted for most of the changes observed through the analytical methods applied.

The use of MAP proved to be a promising method for preserving minimally processed seaweed, surpassing the effectiveness of vacuum packaging in most of the studies. It should be noted that the results obtained from the analyzed seaweeds provide evidence that different species exhibit varied behaviors based on the applied treatments, underscoring the need for the immediate exploration of this preservation technique in other types of marine macroalgae.

The present study displays some limitations, which have been previously discussed. To enhance the understanding of the key compounds that contribute to the distinctive aroma of the seaweeds under investigation, employing techniques such as GC–O and sensory analysis would be of great value. Ideally, in conjunction with conventional cultivation methods, methodologies such as 16S rRNA gene sequencing could aid in identifying the taxonomic composition of the microbiota present. Moreover, expanding the sample size with true replicates and conducting additional repetitions would help to minimize data variability.

The present work represents a first step towards studying minimal processing techniques with the aim of better preserving the organoleptic characteristics of seaweeds. Further research in this area is required, in particular the study of the impact of other MAP gas mixtures, storage temperatures, or lighting settings. Additionally, enhancements in packaging conditions should also be considered; for example, incorporating a nutrient source such as sterilized seawater or exploring the creation of a protective membrane to prevent the seaweeds from experiencing desiccation. Further research to demonstrate the impact of these techniques on the nutritional characteristics, microbial diversity, or acceptance by the consumer market is also important.

## Figures and Tables

**Table 1:** Main VOCs responsible for the variation in *P. umbilicalis* samples according to the PCA.

VOCs	Days	*Porphyra umbilicalis*		
		*CTRL*	*MAP*	*VAC*
*8-Heptadecene (isomer 2)* CAS# N/D 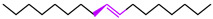 Threshold: N/D	Day 0	10.878 ± 4.982	—	—
	Day 3	7.346 ± 0.801	19.952 ± 1.836	28.141 ± 2.848
	Day 6	12.577 ± 1.852	24.026 ± 3.459	29.272 ± 1.133
	Day 9	10.939 ± 1.969	23.277 ± 2.356	22.992 ± 1.849
	Day 12	12.219 ± 0.368	25.666 ± 1.829	32.619 ± 3.041
	Day 15	14.501 ± 1.913	27.679 ± 3.405	5.527 ± 1.823
*Heptadecane* CAS# 629-78-7 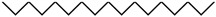 Threshold: N/D	Day 0	3.037 ± 1.524	—	—
	Day 3	0.924 ± 0.024	7.098 ± 0.955	6.817 ± 0.667
	Day 6	1.109 ± 0.058	7.92 ± 1.481	8.552 ± 0.732
	Day 9	0.961 ± 0.290	6.77 ± 0.710	6.648 ± 0.185
	Day 12	1.192 ± 0.048	7.41 ± 0.338	7.271 ± 0.559
	Day 15	2.037 ± 0.262	10.283 ± 0.975	1.143 ± 0.418
*Pentadecanal* CAS# 2765-11-9 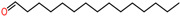 Threshold: 0.715 mg/kg	Day 0	1.826 ± 0.464	—	—
	Day 3	17.544 ± 0.702	8.747 ± 0.709	0.695 ± 0.011
	Day 6	10.309 ± 0.124	25.939 ± 3.018	1.529 ± 0.689
	Day 9	12.627 ± 3.127	25.229 ± 2.091	1.993 ± 0.531
	Day 12	23.097 ± 0.329	19.565 ± 1.615	2.827 ± 0.132
	Day 15	12.968 ± 1.836	11.103 ± 0.539	1.732 ± 0.549

Notes: The results are expressed as the percentage (%) of the respective peak area relative to the total peak area. Odor thresholds in water were calculated using the median for the values compiled by Gemert [59].

**Table 2:** Main VOCs responsible for the variation in *U. lactuca* samples according to the PCA.

VOCs	Days	*Ulva lactuca*		
		*CTRL*	*MAP*	*VAC*
*2,4-Decadienal (isomer)* CAS# 25152-84-5 (?) 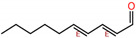 Threshold: 0.00013 mg/kg	Day 0	27.565 ± 0.082	—	—
	Day 3	9.401 ± 0.596	8.748 ± 0.206	10.866 ± 1.603
	Day 6	2.166 ± 0.003	9.163 ± 0.299	9.387 ± 1.902
	Day 9	0.695 ± 0.105	9.796 ± 0.081	2.521 ± 0.002
	Day 12	0.506 ± 0.021	8.731 ± 0.827	6.464 ± 0.024
	Day 15	0.810 ± 0.163	4.250 ± 0.712	6.128 ± 0.142
*2,4-Decadienal, (E,E)-* CAS# 2363-88-4 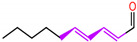 Threshold: 0.0003 mg/kg	Day 0	12.425 ± 0.412	—	—
	Day 3	4.018 ± 0.248	8.110 ± 1.208	6.853 ± 0.463
	Day 6	1.775 ± 0.022	12.590 ± 0.687	8.237 ± 0.381
	Day 9	0.701 ± 0.118	12.584 ± 0.104	5.377 ± 0.004
	Day 12	0.950 ± 0.027	20.548 ± 1.950	10.449 ± 0.038
	Day 15	1.210 ± 0.253	12.312 ± 1.823	11.744 ± 1.109
*1-Hexanol* CAS# 111-27-3 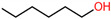 Threshold: 1.21 mg/kg	Day 0	0.133 ± 0.025	—	—
	Day 3	6.025 ± 0.050	0.856 ± 0.053	0.504 ± 0.029
	Day 6	14.377 ± 1.316	1.074 ± 0.070	0.611 ± 0.011
	Day 9	17.684 ± 0.261	0.443 ± 0.004	0.879 ± 0.001
	Day 12	24.474 ± 0.584	1.102 ± 0.103	0.850 ± 0.003
	Day 15	4.670 ± 0.477	0.828 ± 0.030	1.594 ± 0.176
*Benzaldehyde* CAS# 100-52-7 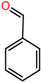 Threshold: 0.55 mg/kg	Day 0	2.287 ± 0.027	—	—
	Day 3	2.939 ± 0.571	4.004 ± 0.207	5.146 ± 0.094
	Day 6	5.586 ± 2.270	4.136 ± 0.325	3.371 ± 1.052
	Day 9	3.804 ± 0.431	8.441 ± 0.070	15.221 ± 0.010
	Day 12	6.023 ± 1.384	11.037 ± 0.812	17.494 ± 0.064
	Day 15	9.642 ± 0.367	33.800 ± 9.784	11.8 ± 2.147
*8-Heptadecene (isomer 1)* CAS# N/D 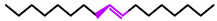 Threshold: N/D	Day 0	6.000 ± 1.107	—	—
	Day 3	13.026 ± 0.003	12.674 ± 1.198	13.193 ± 2.260
	Day 6	15.353 ± 1.827	16.715 ± 0.625	15.777 ± 4.864
	Day 9	16.710 ± 0.912	19.334 ± 0.160	16.086 ± 0.011
	Day 12	6.424 ± 0.752	12.419 ± 1.177	8.505 ± 0.031
	Day 15	8.799 ± 0.376	7.026 ± 2.471	9.521 ± 0.380

Notes: The results are expressed as the percentage (%) of the respective peak area relative to the total peak area. Odor thresholds in water were calculated using the median for the values compiled by Gemert [59].

## Data Availability

Data from the present study are available on request from the Faculty of Science and Technology of Universidade NOVA de Lisboa (FCT-NOVA).

## Supplementary Materials

The following supporting information can be downloaded at: https://www.mdpi.com/article/10.3390/foods12142736/s1. Table S1: Color changes and color simulation throughout storage days for *P. umbilicalis* and *U. lactuca* preserved by different treatments; Table S2: Microbial counts for Coliforms, *E. coli*, Coagulase-positive Staphylococci, *Vibrio* spp., *Salmonella* spp., and *L. monocytogenes* at storage days 0 and 15 for *P. umbilicalis* and *U. lactuca* preserved by different treatments; Table S3a: ANOVA results for the GC-TOF-MS analysis of *Porphyra umbilicalis*; Table S3b: ANOVA results for the GC-TOF-MS analysis of *Ulva lactuca*; Table S3c: Tukey HSD for the GC-TOF-MS analysis of *P. umbilicalis* and *U. lactuca*; Table S4: French guidelines that apply to dry seaweed products and Portuguese guidelines that apply to “minimally processed” food products; Figure S1: Diversity of VOCs over time in *Porphyra umbilicalis* and *Ulva lactuca*, grouped by functional groups; Figure S2: Heatmap graphic with the GC-TOF-MS results for *Porphyra umbilicalis*; Figure S3: Heatmap graphic with the GC-TOF-MS results for *Ulva lactuca*.
