# Supplementary material for: Modified Atmosphere Packaging (MAP) for Seaweed Conservation: Impact on Physicochemical Characteristics and Microbiological Activity
Source: Foods. 2023 Jul 18;12(14):2736. doi: 10.3390/foods12142736 (PMC10379051; doi:10.3390/foods12142736)
Supplement: Supplementary file 1 [file foods-12-02736-s001.zip › foods-2494521-supplementary.pdf]

Supplementary Material

Tables

Table S1. Color changes[#] and color simulation throughout storage days for *P. umbilicalis* and *U. lactuca* preserved by different treatments. 2
Table S2. Microbial counts* for Coliforms, *E. coli*, Coagulase-positive Staphylococci, *Vibrio* spp., *Salmonella* spp., and *L. monocytogenes* at storage days 0 and 15 for *P. umbilicalis* and *U. lactuca* preserved by different treatments. 3
Table S3a. ANOVA results for the GC-TOF-MS analysis of *Porphyra umbilicalis* 4
Table S3b. ANOVA results for the GC-TOF-MS analysis of *Ulva lactuca* 4
Table S3c. Tukey HSD for the GC-TOF-MS analysis of *P. umbilicalis* and *U. lactuca*. 4
Table S4. French guidelines that apply to dry seaweed products and Portuguese guidelines that apply to "minimally processed" food products [1,2]. 5

Figures

Figure S1. Diversity of VOCs over time in *Porphyra umbilicalis* and *Ulva lactuca*, grouped by functional groups. 6
Figure S2. Heatmap graphic with the GC-TOF-MS results for *Porphyra umbilicalis* 7
Figure S3. Heatmap graphic with the GC-TOF-MS results for *Ulva lactuca* 8

Table S1. Color changes# and color simulation throughout storage days for *P. umbilicalis* and *U. lactuca* preserved by different treatments.

The results are expressed as the CIELAB coordinates for *L**, *a**, *b**, and the calculated color deviation (*ΔE*).

**CIELAB Coordinates**	**Days**	***Porphyra umbilicalis* (PU)**			***Ulva lactuca* (UL)**		
		CTRL	*MAP*	*VAC*	*CTRL*	*MAP*	*VAC*
*L**	*Day 0*	32.18 $^{a, A}$ [30.60, 35.54]	32.18 $^{a, A}$ [30.60, 35.54]	32.18 $^{a, A}$ [30.60, 35.54]	73.59 $^{a, A}$ [71.99, 76.84]	73.59 $^{a, A}$ [71.99, 76.84]	73.59 $^{ab, A}$ [71.99, 76.84]
	Day 3	36.84 $^{a, A}$ [34.73, 37.24]	35.24 $^{ab, A}$ [34.00, 37.1]	34.47 $^{a, A}$ [33.82, 35.65]	76.58 $^{ab, A}$ [76.38, 78.41]	77.56 $^{ab, A}$ [75.75, 77.97]	71.41 $^{b, B}$ [70.46, 74.79]
	Day 6	33.79 $^{a, A}$ [33.07, 35.53]	37.38 $^{b, A}$ [34.8, 38.89]	36.38 $^{ab, A}$ [33.38, 39.16]	78.80 $^{bc, A}$ [77.11, 80.39]	77.35 $^{ab, A}$ [75.68, 78.8]	78.08 $^{ac, A}$ [76.63, 80.15]
	Day 9	36.55 $^{a, A}$ [34.93, 38.77]	34.38 $^{ab, A}$ [30.42, 35.95]	34.35 $^{a, A}$ [31.32, 39.21]	76.27 $^{ab, AB}$ [75.87, 77.82]	78.37 $^{b, A}$ [77.62, 79.23]	74.02 $^{b, B}$ [69.85, 76.86]
	Day 12	36.73 $^{a, A}$ [35.29, 39.00]	37.42 $^{b, A}$ [35.45, 40.57]	41.00 $^{b, A}$ [39.41, 45.03]	77.02 $^{ab, A}$ [73.18, 78.52]	77.71 $^{ab, A}$ [75.82, 78.46]	78.48 $^{c, A}$ [77.16, 79.6]
	Day 15	36.55 $^{a, A}$ [36.00, 37.28]	36.51 $^{ab, A}$ [34.38, 39.72]	42.61 $^{b, A}$ [39.55, 43.19]	81.41 $^{c, A}$ [79.88, 81.86]	77.03 $^{ab, B}$ [73.85, 77.74]	79.32 $^{c, A}$ [78.25, 80.68]
*a**	*Day 0*	6.72 $^{a, A}$ [6.19, 8.32]	6.72 $^{a, A}$ [6.19, 8.32]	6.72 $^{ac, A}$ [6.19, 8.32]	-14.73 $^{a, A}$ [-16.50, -13.82]	-14.73 $^{a, A}$ [-16.50, -13.82]	-14.73 $^{a, A}$ [-16.50, -13.82]
	Day 3	5.18 $^{ab, A}$ [4.20, 5.52]	3.73 $^{b, A}$ [2.84, 4.94]	4.59 $^{b, A}$ [3.97, 5.69]	-13.87 $^{ab, A}$ [-14.95, -13.16]	-12.70 $^{b, A}$ [-13.37, -11.93]	-14.72 $^{a, A}$ [-16.02, -13.53]
	Day 6	3.63 $^{b, A}$ [3.09, 4.28]	4.68 $^{bc, A}$ [2.65, 5.28]	4.06 $^{b, A}$ [3.34, 4.93]	-14.04 $^{ab, A}$ [-14.76, -13.57]	-11.49 $^{bc, B}$ [-12.14, -10.93]	-10.36 $^{bc, B}$ [-11.20, -10.18]
	Day 9	5.17 $^{ab, A}$ [4.54, 6.34]	6.35 $^{ac, A}$ [5.31, 7.68]	5.41 $^{ab, A}$ [4.30, 6.69]	-13.02 $^{ab, A}$ [-14.03, -12.37]	-10.08 $^{c, B}$ [-10.79, -9.44]	-10.73 $^{b, B}$ [-12.04, -10.46]
	Day 12	3.58 $^{b, A}$ [3.22, 4.39]	9.19 $^{a, B}$ [7.67, 10.70]	8.36 $^{c, B}$ [7.55, 13.20]	-12.93 $^{b, A}$ [-13.72, -12.67]	-10.74 $^{c, B}$ [-11.93, -8.64]	-10.64 $^{bc, B}$ [-11.31, -9.97]
	Day 15	4.18 $^{b, A}$ [3.66, 5.28]	7.27 $^{a, B}$ [5.72, 9.87]	13.25 $^{c, B}$ [10.17, 15.35]	-10.58 $^{c, A}$ [-11.31, -9.50]	-9.17 $^{c, A}$ [-10.88, -8.78]	-8.43 $^{c, A}$ [-9.16, -7.96]
*b**	*Day 0*	27.74 $^{a, A}$ [26.85, 28.41]	27.74 $^{a, A}$ [26.85, 28.41]	27.74 $^{a, A}$ [26.85, 28.41]	44.61 $^{a, A}$ [43.59, 47.05]	44.61 $^{a, A}$ [43.59, 47.05]	44.61 $^{ab, A}$ [43.59, 47.05]
	Day 3	24.07 $^{a, A}$ [22.81, 27.04]	23.55 $^{abc, A}$ [22.22, 25.99]	24.01 $^{ab, A}$ [22.84, 26.63]	45.76 $^{a, A}$ [39.3, 47.83]	48.97 $^{a, AB}$ [40.98, 50.71]	55.44 $^{b, B}$ [51.08, 56.86]
	Day 6	24.67 $^{a, A}$ [23.60, 26.08]	26.56 $^{ab, A}$ [24.93, 27.30]	25.31 $^{ab, A}$ [24.10, 26.34]	35.42 $^{ab, A}$ [32.46, 43.8]	42.32 $^{ab, A}$ [40.53, 47.29]	37.44 $^{ac, A}$ [32.94, 47.00]
	Day 9	26.97 $^{a, A}$ [24.64, 28.4]	24.66 $^{bc, AB}$ [23.48, 25.26]	22.59 $^{bc, B}$ [21.93, 23.18]	41.15 $^{a, A}$ [32.36, 45.64]	35.71 $^{b, A}$ [32.34, 41.59]	49.49 $^{b, B}$ [47.40, 53.18]
	Day 12	26.49 $^{a, A}$ [25.80, 28.26]	22.33 $^{c, B}$ [20.49, 23.88]	19.62 $^{bc, B}$ [2.67, 24.84]	41.80 $^{a, A}$ [38.60, 44.68]	35.90 $^{ab, A}$ [34.96, 49.28]	37.06 $^{ac, A}$ [35.10, 40.44]
	Day 15	27.13 $^{a, A}$ [25.33, 29.44]	19.31 $^{bc, B}$ [11.19, 25.17]	1.68 $^{c, B}$ [0.86, 8.39]	27.38 $^{b, A}$ [25.94, 28.57]	46.76 $^{a, B}$ [41.66, 49.3]	34.83 $^{c, A}$ [32.75, 36.76]
ΔE	*Day 0*	–	–	–	–	–	–
	Day 3	6.19 $^{a, A}$ [5.12, 8.19]	7.21 $^{a, A}$ [5.06, 8.48]	5.24 $^{a, A}$ [3.45, 6.86]	4.65 $^{a, A}$ [3.62, 7.36]	6.64 $^{a, A}$ [6.05, 7.27]	10.62 $^{a, A}$ [8.69, 12.6]
	Day 6	5.35 $^{a, A}$ [3.80, 6.07]	6.49 $^{a, A}$ [4.87, 8.25]	5.79 $^{ab, A}$ [4.58, 8.13]	10.89 $^{a, A}$ [4.92, 14.56]	6.59 $^{a, A}$ [5.45, 7.72]	11.4 $^{a, A}$ [7.62, 14.41]
	Day 9	4.97 $^{a, A}$ [2.87, 7.26]	4.69 $^{a, A}$ [4.03, 6.34]	6.85 $^{ab, A}$ [5.80, 8.85]	6.12 $^{a, A}$ [4.20, 13.58]	11.34 $^{a, A}$ [7.73, 15.38]	7.80 $^{a, A}$ [6.05, 10.08]
	Day 12	5.16 $^{a, A}$ [4.92, 7.13]	7.56 $^{a, A}$ [5.76, 11.48]	10.86 $^{bc, A}$ [6.90, 29.03]	5.91 $^{a, A}$ [4.14, 9.86]	11.40 $^{a, A}$ [7.98, 12.86]	10.33 $^{a, A}$ [8.11, 12.14]
	Day 15	5.48 $^{a, A}$ [4.92, 6.08]	9.26 $^{a, A}$ [4.00, 17.69]	28.77 $^{c, B}$ [20.66, 29.95]	19.66 $^{b, A}$ [18.05, 21.53]	8.14 $^{a, B}$ [7.78, 9.35]	13.11 $^{a, AB}$ [12.06, 15.51]
Color Simulation	*Day 0*						
	Day 3						
	Day 6						
	Day 9						
	Day 12						
	Day 15						

Notes: Medians and interquartile range (IQR) [Q1, Q3] from duodecuple determinations on each experiment. Medians in the same column followed by a different lowercase letter are significantly different ($P < 0.05$) throughout storage days. Medians in the same row followed by a different uppercase letter are significantly different ($P < 0.05$) among treatments.

Table S2. Microbial counts* for Coliforms, *E. coli*, Coagulase-positive Staphylococci, *Vibrio* spp., *Salmonella* spp., and *L. monocytogenes* at storage days 0 and 15 for *P. umbilicalis* and *U. lactuca* preserved by different treatments. The results are expressed as the logarithm of colony-forming unit per gram of sample (log CFU.g^{-1}).

Microbiological Analysis	***Porphyra umbilicalis* (PU)**				***Ulva lactuca* (UL)**			
	Day 0 CTRL	*Day 15 CTRL*	*Day 15 MAP*	*Day 15 VAC*	*Day 0 CTRL*	*Day 15 CTRL*	*Day 15 MAP*	*Day 15 VAC*
Coliforms	< LOD	< LOD	< LOD	< LOD	< LOD	< LOD	< LOD	< LOD
Escherichia coli	< LOD	< LOD	< LOD	< LOD	< LOD	< LOD	< LOD	< LOD
Coagulase-positive Staphylococci#	2.7637	2.6505	< LOD	< LOD	< LOD	< LOD	< LOD	< LOD
Vibrio spp.	< LOD	< LOD	< LOD	< LOD	< LOD	< LOD	< LOD	< LOD
Salmonella spp.	N/D	N/D	N/D	N/D	N/D	N/D	N/D	N/D
Listeria monocytogenes	N/D	N/D	N/D	N/D	N/D	N/D	N/D	N/D

* Notes: LOD = Limit of Detection; N/D = Not Detected; *Day 0* (n = 2 petri dishes x 2 dilutions x 4 repetitions = 16); *Day 15* (n = 2 petri dishes x 2 dilutions x 1 repetition = 4, for each treatment); LOD < 1 log CFU.g^{-1} for coliforms and *Escherichia coli*, and LOD < 2 log CFU.g^{-1} for Coagulase-positive Staphylococci and *Vibrio* spp.

# Observation: Black colonies without halo, non-characteristic of coagulase-positive Staphylococci.

Analysis of Variance of Aligned Rank Transformed Data

Table Type: ANOVA Table (Type III tests)
Model: No Repeated Measures (LM)
Response: art(Values)
Equation: Values ~ Days + Treatments + Days:Treatments

Table S3a. ANOVA results for the GC-TOF-MS analysis of *Porphyra umbilicalis*

	Variables	Df	Df.res	F value	Pr(>F)	Signif.
1	Days	5	2250	1.87202	0.096001	.
2	Treatments	2	2250	0.39675	0.672550	
3	Days:Treatments	10	2250	0.28115	0.985454	

Notes: Signif. codes: 0 '***' 0.001 '**' 0.01 '*' 0.05 '.' 0.1 ' ' 1

Table S3b. ANOVA results for the GC-TOF-MS analysis of *Ulva lactuca*

	Variables	Df	Df.res	F value	Pr(>F)	Signif.
1	Days	5	2646	18.9221	< 2.22e-16	***
2	Treatments	2	2646	14.4729	5.6058e-07	***
3	Days:Treatments	10	2646	7.3507	1.3834e-11	***

Notes: Signif. codes: 0 '***' 0.001 '**' 0.01 '*' 0.05 '.' 0.1 ' ' 1

Table S3c. Tukey HSD for the GC-TOF-MS analysis of *P. umbilicalis* and *U. lactuca*.

**VOCs**	***Porphyra umbilicalis* (PU)**			***Ulva lactuca* (UL)**		
	CTRL	*MAP*	*VAC*	*CTRL*	*MAP*	*VAC*
Day 0	a, A	a, A	a, A	a, A	a, A	a, A
Day 3	ab, A	ab, A	ab, A	a, A	b, B	b, B
Day 6	b, A	b, A	b, A	a, A	b, B	b, B
Day 9	ab, A	ab, A	ab, A	a, A	b, B	b, B
Day 12	ab, A	ab, A	ab, A	a, A	b, B	b, B
Day 15	ab, A	ab, A	ab, A	a, A	b, B	b, B

* Notes: Different lowercase letters in the same column are significantly different (P < 0.05) throughout storage days. Different uppercase letters in the same row are significantly different (P < 0.05) among treatments (for each seaweed).

Table S4. French guidelines that apply to dry seaweed products and Portuguese guidelines that apply to "minimally processed" food products [1,2].

**Microorganisms / Pathogens**	**French Guidelines Limits (CFU.g^{-1})**	**Portuguese Guidelines Limits (CFU.g^{-1})**
Aerobe mesophiles (30 °C)	$\leq 10^5$	$\leq 10^6$
Yeasts	*****	$\leq 10^5$
Molds	*****	$\leq 5x10^2$
Enterobacteriaceae (37 °C)	*****	$\leq 10^5$
Coliforms (fecal) / *E. coli*	≤ 10	≤ 10 (N/D)
Anaerobe Sulfite Reducers	$\leq 10^2$	*****
Coagulase-positive Staphylococci / *S. aureus*	$\leq 10^2$	≤ 10 (N/D)
Bacillus spp.	*****	$\leq 10^4$
Bacillus cereus	*****	$\leq 10^3$
Clostridium perfringens	≤ 1	$\leq 10^2$
Listeria spp.	*****	≤ 10 (N/D)
Listeria monocytogenes	*****	Not detected (per 25 g)
Salmonella spp.	Not detected (per 25 g)	Not detected (per 25 g)
Vibrio cholerae, *V. parahaemolyticus*, *Campylobacter* spp., *Cronobacter* spp., *Shigella* spp., *Yersinia enterocolitica*	*****	Not detected (per 25 g)

1. CEVA *Macroalgues et Microalgues Alimentaires - Statut Règlementaire En France et En Europe (Synthèse CEVA 2019)*; Pleubian, France, 2019;
2. INSA *Interpretação de Resultados de Ensaios Microbiológicos Em Alimentos Prontos Para Consumo e Em Superfícies Do Ambiente de Preparação e Distribuição Alimentar (Valores-Guia)*; Instituto Nacional de Saúde Doutor Ricardo Jorge: Lisboa, Portugal, 2019;

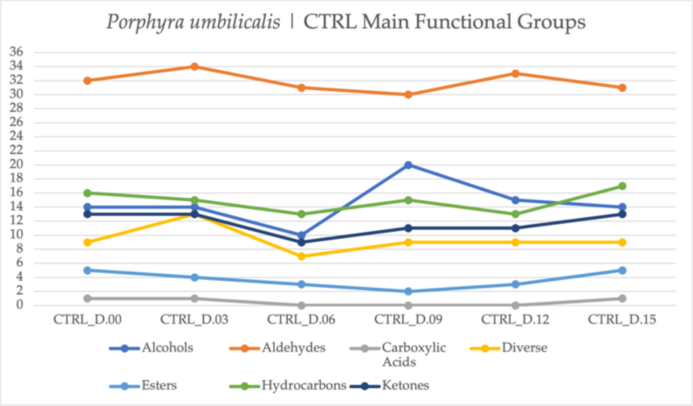

(**a**) Main functional groups of PU-CTRL

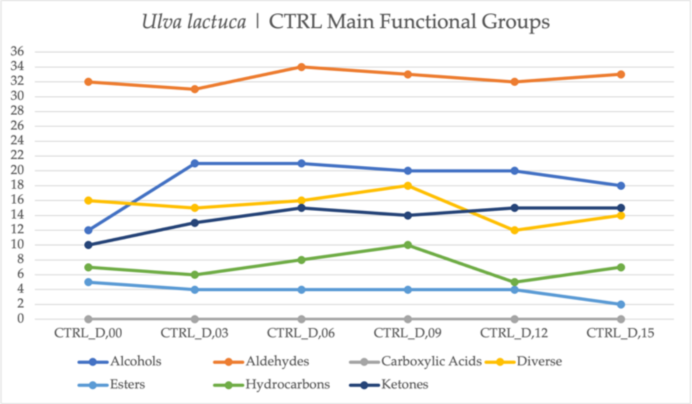

(**b**) Main functional groups of UL-CTRL

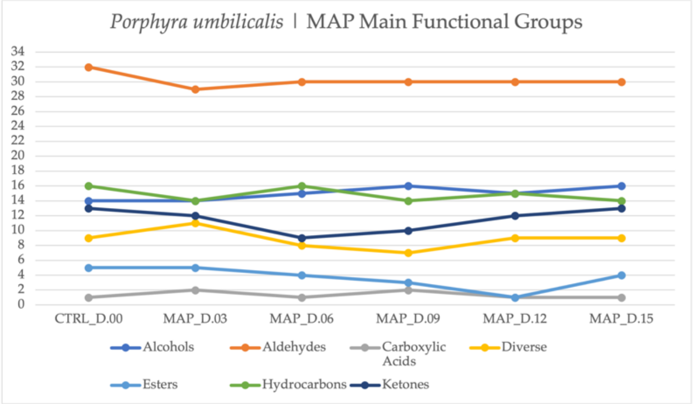

(**c**) Main functional groups of PU-MAP

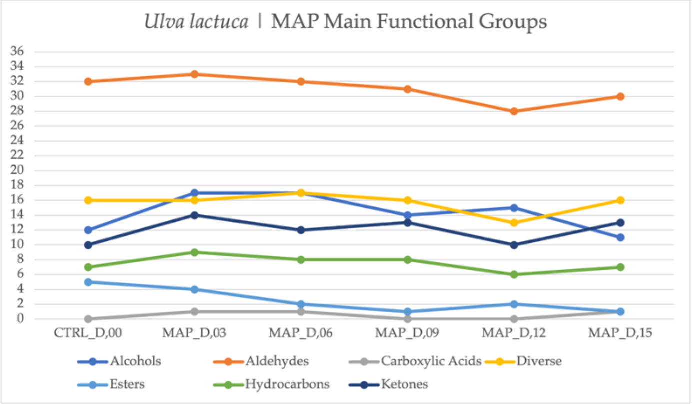

(**d**) Main functional groups of UL-MAP

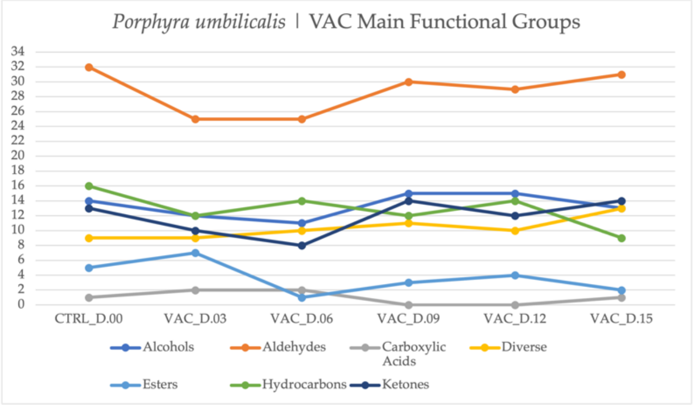

(**e**) Main functional groups of PU-VAC

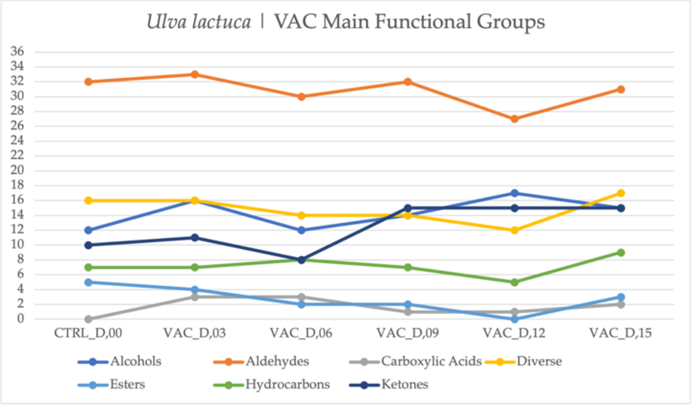

(**f**) Main functional groups of UL-VAC

Figure S1. Diversity of VOCs over time in *Porphyra umbilicalis* and *Ulva lactuca*, grouped by functional groups.

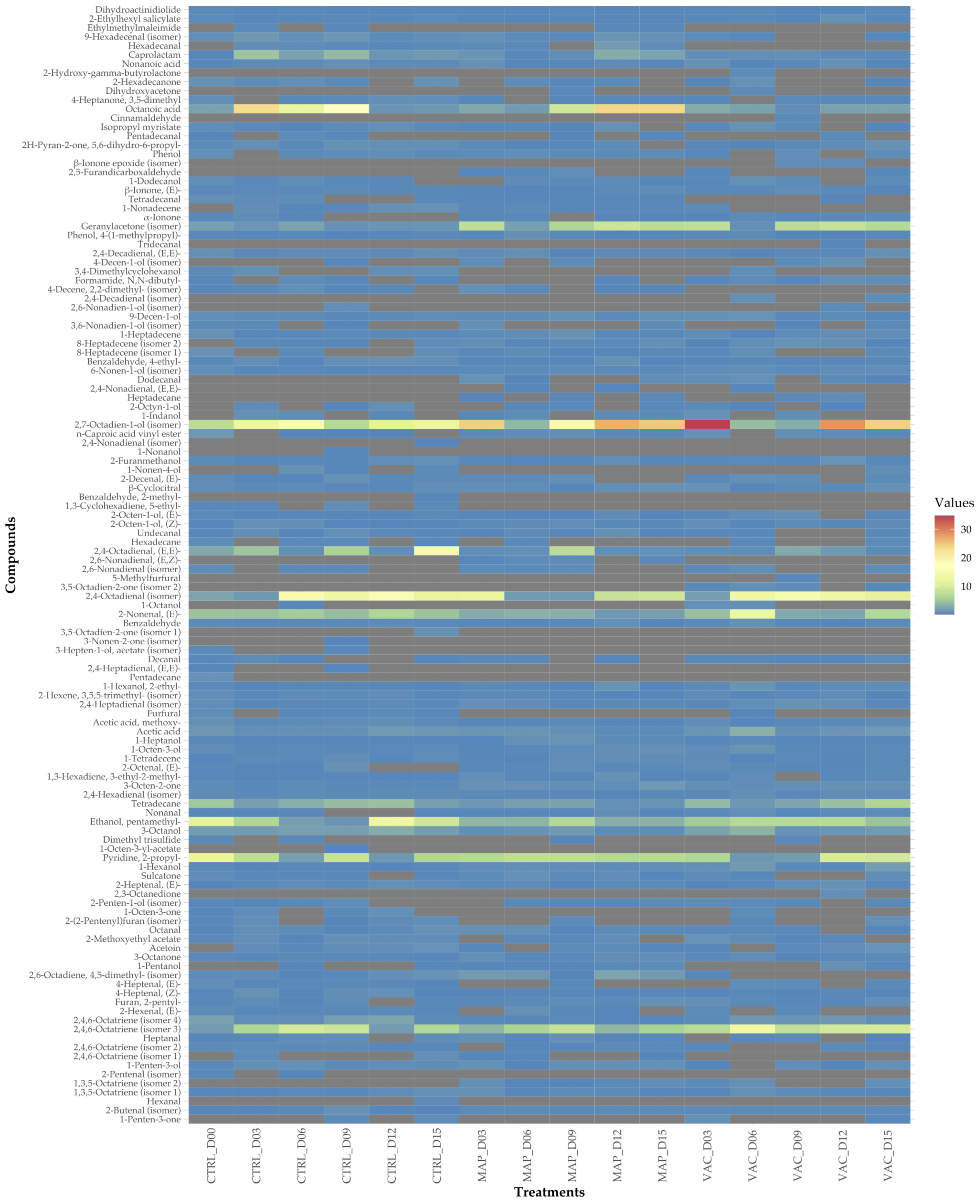

Figure S2. Heatmap graphic with the GC-TOF-MS results for *Porphyra umbilicalis*

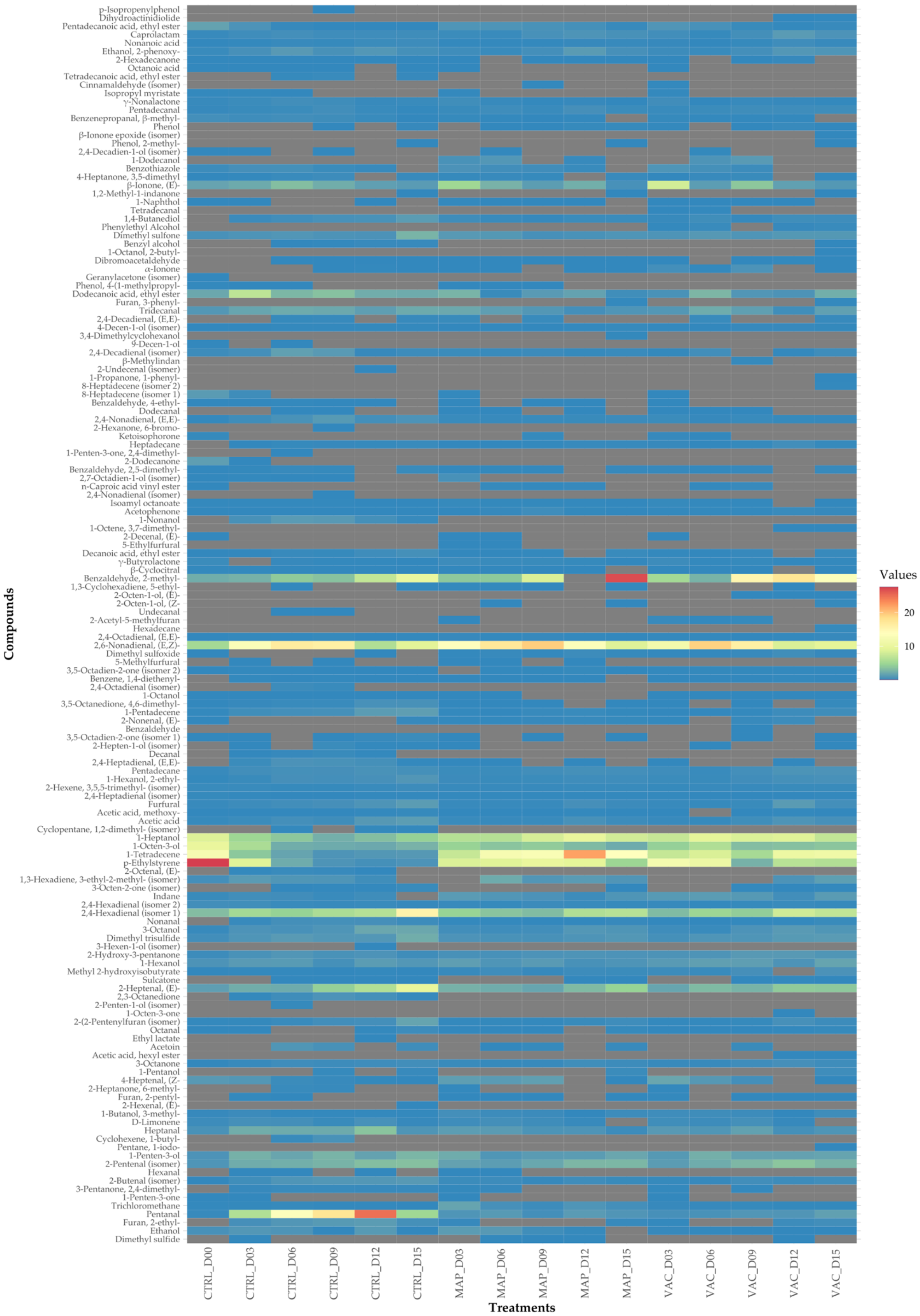

Figure S3. Heatmap graphic with the GC-TOF-MS results for *Ulva lactuca*
